# *De novo* Sequencing and Transcriptome Analysis of *Pinellia ternata* Identify the Candidate Genes Involved in the Biosynthesis of Benzoic Acid and Ephedrine

**DOI:** 10.3389/fpls.2016.01209

**Published:** 2016-08-16

**Authors:** Guang-hui Zhang, Ni-hao Jiang, Wan-ling Song, Chun-hua Ma, Sheng-chao Yang, Jun-wen Chen

**Affiliations:** Yunnan Research Center on Good Agricultural Practice for Dominant Chinese Medicinal Materials, Yunnan Agricultural UniversityKunming, China

**Keywords:** *Pinellia ternata*, transcriptome, phenylpropylamino alkaloids, ephedrine, *De novo* sequencing

## Abstract

**Background:** The medicinal herb, *Pinellia ternata*, is purported to be an anti-emetic with analgesic and sedative effects. Alkaloids are the main biologically active compounds in *P. ternata*, especially ephedrine that is a phenylpropylamino alkaloid specifically produced by *Ephedra* and *Catha edulis*. However, how ephedrine is synthesized in plants is uncertain. Only the phenylalanine ammonia lyase (PAL) and relevant genes in this pathway have been characterized. Genomic information of *P. ternata* is also unavailable.

**Results:** We analyzed the transcriptome of the tuber of *P. ternata* with the Illumina HiSeq™ 2000 sequencing platform. 66,813,052 high-quality reads were generated, and these reads were assembled *de novo* into 89,068 unigenes. Most known genes involved in benzoic acid biosynthesis were identified in the unigene dataset of *P. ternata*, and the expression patterns of some ephedrine biosynthesis-related genes were analyzed by reverse transcription quantitative real-time PCR (RT-qPCR). Also, 14,468 simple sequence repeats (SSRs) were identified from 12,000 unigenes. Twenty primer pairs for SSRs were randomly selected for the validation of their amplification effect.

**Conclusion:** RNA-seq data was used for the first time to provide a comprehensive gene information on *P. ternata* at the transcriptional level. These data will advance molecular genetics in this valuable medicinal plant.

## Introduction

*Pinellia ternata* (Thunb.) Berit. is an anti-emetic with analgesic and sedative effects, and has been applied as an antitussive and expectorant (He et al., 2005; Wang and Zhou, 2007; Zhang et al., 2007). The dried tuber of this herb, called “banxia” in Chinese, is in the top 10 most commonly used traditional Chinese medicines. The alkaloids isolated from the tubers of *P. ternata* are said to have anticancer properties (Wang and Zhou, 2007). *P. ternata* is widely distributed in China and other Asian countries. Due to overexploitation and lack of large-scale plantings, *P. ternata* sources are becoming increasingly scarce.

Secondary metabolites of *P. ternata* have been identified: alkaloids (main active ingredient; i.e., ephedrine), organic acids, volatile oils, sterols, and amino acids (Oshio et al., 1978; Masao, 1997; Ge and Wu, 2009). Ephedrine accumulates primarily in mature tubers and is of great interest to researchers (Wu et al., 1996; Xu et al., 2007). Ephedrine and other phenylpropylamino alkaloids, such as, (*1S, 2S*)-pseudoephedrine, (*S*)-cathinone, (*1R, 2S*)-norephedrine, and (*1S, 2S*)-pseudonorephedrine are particularly produced by plants in the genus *Ephedra* and by *Catha edulis*. The US Food and Drug Administration has banned ephedra-containing supplements. In addition, dietary supplements that contain ephedrine are illegal in the United States for its serious side effects. Despite these limitations, ephedrine still is listed on the WHO Model List of Essential Medicines, and has been used to prevent low blood pressure, asthma, narcolepsy, and obesity. This motivates efforts to increase the production of ephedrine in planta. Further, to obtain purified extracts of ephedrine from plant, it is paramount to understand the structurally-related metabolites on the ephedrine pathway. This knowledge will provide information on potentially unforeseen byproducts and on how intermediates on the pathway can be chemically separated by chromatographic techniques. Therefore, a complete understanding of the biosynthesis of phenylpropylamino alkaloids still needs to be fully elucidated (Groves et al., 2015).

**Figure 1:**
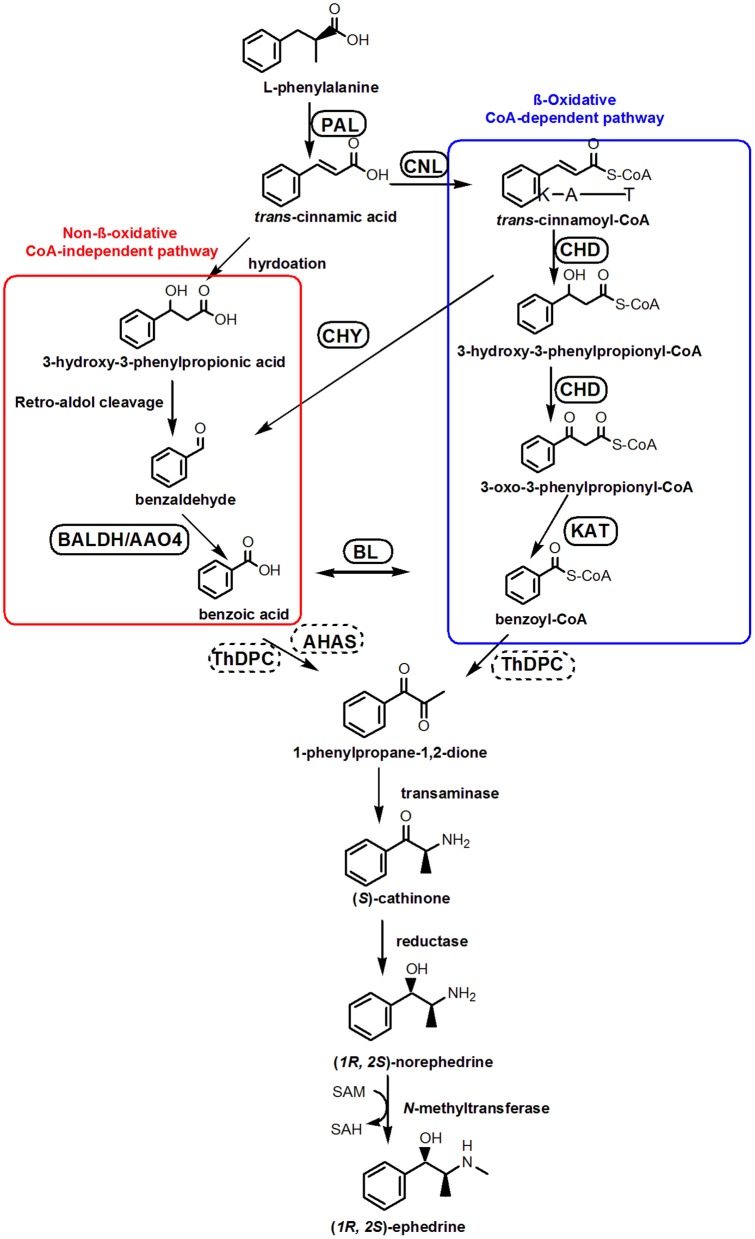
**Proposed pathways for the biosynthesis of ephedrine in *P. ternata***. A CoA-independent, non-β-oxidative pathway of L-phenylalanine side-chain shortening is shown in red boxes, whereas a CoA-dependent, β-oxidative route is shown in blue boxes. Abbreviations: PAL, phenylalanine ammonia lyase; CNL, cinnamate:CoA ligase; CHD, cinnamoyl-CoA hydratase-dehydrogenase; CHY, 3-hydroxyisobutyryl-CoA hydrolase; AO4, aldehyde oxidases 4; KAT, 3-ketoacyl-CoA thiolase; BALDH, benzaldehyde dehydrogenase; BL, benzoate-CoA ligase; ThPDC, ThDP-dependent pyruvate decarboxylase; AHAS, acetolactate synthase.

Phenylpropylamino alkaloid biosynthesis begins with L-phenylalanine (Phe) (Hagel et al., 2011) which is deaminated by phenylalanine ammonia lyase (PAL) (Soerensen and Spenser, 1994). Recent studies suggest that Phe-derived benzoic acid is an intermediate in the formation of phenylpropylamino alkaloids (Krizevski et al., 2010), although the involvement of benzoyl-CoA or benzaldehyde cannot be ruled out. There are at least two possible pathways of Phe side-chain shortening in plant benzoic acid biosynthesis: β-oxidative and non-β-oxidative routes (Boatright et al., 2004). A proposed biosynthesis pathway for benzoic acid and ephedrine synthesis is depicted as in Figure 1 (Facchini, 2001; Long et al., 2009; Krizevski et al., 2012a,b).

In the β-oxidative pathway, the first step is cinnamoyl-CoA formation, catalyzed by cinnamate: CoA ligase (CNL) (Gaid et al., 2012; Klempien et al., 2012), which was also called as acyl activating enzyme (AAE) (Colquhoun et al., 2012). Subsequently, a bifunctional peroxisomal enzyme (cinnamoyl-CoA hydratase-dehydrogenase, CHD) converting cinnamoyl-CoA to 3-oxo-3-phenylpropanoyl-CoA, and 3-ketoacyl-CoA thiolase (KAT) catalyzing the formation of benzoyl-CoA (Figure 1; Van et al., 2009). In the non-β-oxidative pathway, two distinct dehydrogenase catalyzing the formation of benzoic acid from benzaldehyde have been characterized, and their genes, benzaldehyde dehydrogenase (*BALDH*) gene from *Antirrhinum majus* (Long et al., 2009) and aldehyde oxidases 4 (*AO4*) gene from *Arabidopsis thaliana* (Ibdah et al., 2009) have been cloned. After the formation of benzoic acid, the synthesis of phenylpropylamino alkaloid is initiated by condensation of pyruvic acid and benzoic acid to form 1-phenylpropane-1,2-dione (Figure 1). The enzyme that catalyzes this reaction has not been identified, but a ThDP-dependent pyruvate decarboxylase (ThPDC) or an acetolactate synthase (AHAS) have been suggested (Müller et al., 2009).

RNA-sequencing (RNA-seq) is a particularly effective technology for gene discovery, especially in non-model species for which reference genome sequences are not available. As mentioned above, the biosynthesis of ephedrine and other phenylpropylamino alkaloids is still largely unknown. Recently, candidate genes potentially involved in phenylpropylamino alkaloids biosynthesis in *C. edulis* and *E. sinica* were revealed using Illumina next-generation sequencing (NGS) (Groves et al., 2015). Here, we characterized the transcriptome of the tuber of *P. ternata* and identified candidate genes that encode enzymes in the ephedrine biosynthetic pathway. Based on the transcriptome sequences, SSR markers were predicted in *P. ternata* to facilitate molecular genetics in this valuable medicinal plant.

## Materials and methods

### Ethics statement

No specific permits were required for the described field studies. No specific permissions were required for these locations and activities. The location is not privately-owned or protected in any way and the field studies did not involve endangered or protected species.

### Plant material

*P. ternata* was cultivated in experimental fields at Purui Bio Pharmaceutical Co., Ltd., located in Shizong County, Yunnan province, southwest of China (24° 46′ 40″N, 104° 5′ 34″E, alt. 1886 m). *P. ternata* tubers ~1.5–2.0 cm in diameter were harvested from 1-year-old plants (Data Sheet 1: Figure S1). Tubers were collected and immediately frozen in liquid nitrogen and stored at −80°C until use.

### cDNA library construction and sequencing

Total RNA was extracted from the mature tubers using the Trizol Kit (Promega, USA) according to the manufacturer's instructions, and poly (A) mRNA was purified from 20 μg of total RNA using Oligo (dT) magnetic beads. Subsequently, mRNA was fragmented into smaller pieces (200–700 bp), which were used for first-strand cDNA synthesis with reverse transcriptase and random hexamer-primer. Subsequently, second-strand cDNA was synthesized using buffer, dNTPs, RNaseH, and DNA polymerase I. The short double-stranded cDNA fragments were purified with QiaQuick PCR extraction kit and resolved with EB buffer. These cDNA fragments underwent an end-repair process and poly(A) was added and then ligated with the Illumina paired-end sequencing adaptors. Ligation products were purified with magnetic beads and separated by agarose gel electrophoresis. A range of cDNA fragments (200 ± 25 bp) were excised from the gel and selected for PCR amplification as templates. The cDNA library was constructed with a fragment-length range of 200 bp (±25 bp). Finally, the cDNA libraries were sequenced on a paired-end flow cell using an Illumina HiSeq™ 2000 at Genedenovo Bio-Tech Co., Ltd (Guangzhou, China). The dataset of high-quality reads was deposited in a NCBI database under accession number SRX484200.

### *De novo* transcripts assembly

The image data output from the sequencing machine was transformed by base calling into sequence data (raw data/reads). Raw reads are transformed into clean reads by removing reads with sequencing adaptors; removing reads with frequency of unknown nucleotides above 5%; and removing low-quality reads (containing more than 50% bases with *Q* ≤ 20) using a custom Perl script. Transcripts *de novo* assembly was carried out using two short read assembly programs: Trinity (Grabherr et al., 2011) and Bridger (Chang et al., 2015). Clean reads were *de novo* assembled with Trinity with the fixed default k-mer size of 25. Trinity initially combines reads with certain length of overlap to form longer fragments without N (using “N” to represent unknown sequences), or contigs. Then, contigs are processed with sequence clustering software TIGR Gene Indices clustering tools (TGICL) (Pertea et al., 2003) to form longer sequences without N and these sequences are defined as unigenes.

Finally, all assembled unigenes were searched using BLASTX against protein databases, such as non-redundant (NR) protein database (http://www.ncbi.nlm.nih.gov/), Swiss-Prot database (http://www.expasy.ch/sprot), the Kyoto Encyclopedia of Genes and Genomes (KEGG) pathway database (http://www.genome.jp/kegg) (Kanehisa et al., 2006), and the Cluster of Orthologous Groups (COG) (Tatusov et al., 1997) database (http://www.ncbi.nlm.nih.gov/COG/), with an *E*-value cutoff of 1e-5. The best match from the four databases was used to decide unigene sequence direction. If database information was conflicting, a priority order of NR, SwissProt, KEGG, and COG was followed when deciding unigene sequence direction. When a unigene would not align to any database, unigene orientation was predicted using ESTScan (Iseli et al., 1999). We also plotted the ratio of assembled unigene length to *Oryza sativa* ortholog length against coverage depth for assessing the extent of transcript coverage provided by unigenes and to evaluate how coverage depth affected unigenes assembly. Firstly, we compared the sequences of all unigenes by BLASTX against the Nr database and found *O. sativa* is one of the top-hit species, which is also monocot as same as *P. ternata*. The *Oryza sativa* orthologs, coverage, and their CDS regions were also obtained in NCBI.

### Functional annotation and predicted CDS and identification of transcription factors

In functional annotation, all assembled unigenes were searched against the NR, Swiss-Prot, KEGG, and COG databases using BLASTN (*E* < 10^−5^) to predict possible functional classifications and molecular pathways. All unigenes were annotated by BLASTX (*E* < 10^−5^) against the *Arabidopsis* TAIR10 peptide database (Swarbreck et al., 2008). Moreover, the conserved domains and families of the assembled unigenes encoding proteins were searched against the Pfam database (version 26.0) (http://pfam.xfam.org/) using the Pfam_Scan program (Finn et al., 2014). To obtain the final functional annotation of the unigenes, the best annotation was chosen based on the BLASTX scores (Camacho et al., 2009). If a unigene did not have annotations in any of the above-mentioned databases, Pfam annotation was assigned to the unigene. Based on the results from NR database annotation, the Blast2GO program (Conesa et al., 2005) was used to obtain GO unigene annotations. Then, WEGO software (Ye et al., 2006) was used to perform GO functional classification for all unigenes to view gene function distribution.

The unigenes coding sequence (CDS) was predicted by Blastx and ESTscan. The unigene sequences were first aligned with the protein databases using BLASTX (*E* < 10^−5^) in the following order: NR, SwissProt, KEGG, and COG. Unigenes aligned to a higher priority database were not aligned to lower priority databases. The best alignment results were used to determine the unigenes sequence directions. Unigene oorientation and CDS with no hits in Blast were predicted using ESTScan (Iseli et al., 1999). To identify the transcription factors, all unigenes were searched against PlnTFDB database (Pérez-Rodríguez et al., 2010) using iTAK analysis tool (http://bioinfo.bti.cornell.edu/cgi-bin/itak/index.cgi) (Dai et al., 2013).

### RT-qPCR analysis

Total RNA from tubers and leaves of *P. ternata* were extracted individually using Trizol Kit (Promega, USA) following the manufacturer's protocol. Subsequently, RNA was treated with 4 × gDNA wiperMix at 42°C for 2 min to remove DNA. The purified RNA (1 ug) was reverse transcribed to cDNA using HiScript QRT SuperMix for qPCR (Vazyme, Nanjing, China). The qPCR reactions were performed in a 20 μl volume composed of 2 μl of cDNA, 0.4 μl of each primer, and 10 μl 2 × SYBR Green Master mix (TaKaRa) in Roche LightCycler 2.0 system (Roche Applied Science, Branford, CT). PCR amplification was performed under the following conditions: 3 min at 94°C, followed by 45 cycles of 94°C for 20 s, 55°C for 20 s, and 72°C for 20 s. Three technical replications were performed for all quantitative PCRs. The glyceraldehyde-3-phosphate dehydrogenase (GAPDH) was chosen as reference gene control for normalization. The relative changes in gene expression levels were calculated using the 2^−ΔΔCt^ method (Livak and Schmittgen, 2001). The log2 value of 2^−ΔΔCt^ was used for representing the relative expressions of each gene. All primers used for the reverse transcription quantitative real-time PCR (RT-qPCR) assay are listed in Data Sheet 1: Table S1.

### SSR detection and validation

Simple sequence repeats (SSRs) were identified using the MIcroSAtellite Identification Tool (http://pgrc.ipk-gatersleben.de/misa/misa.html). Parameters were designed for identifying di-, tri-, tetra-, penta- and hexa-nucleotide motifs with a minimum of 6, 5, 4, 4, and 4 repeats, respectively (Zeng et al., 2010). A maximum distance of 100 nucleotides was allowed between two SSRs. Primer3 (http://primer3.ut.ee/) was employed to design PCR primers flanking each unique SSR region that was identified.

A total of 20 primer pairs (Data Sheet 1: Table S2) were randomly selected to evaluate their amplification effect. The method was performed as our previously described (Jiang et al., 2014), but with the following modification: The PCR reactions were performed at 94°C for 5 min, and followed by 30 cycles of 1 min at 94°C, 50 s at Tm (annealing temperature), 90 s at 72°C and a final step at 72°C for 10 min.

## Results

### Illumina paired-end sequencing and *De novo* assembly

To obtain an overview of the transcriptome of *P. ternata*, a cDNA library was generated from total RNA of mature tubers, and pair-end sequenced using the Illumina HiSeq™ 2000 sequencing platform. After the removal of adaptor, sequences, ambiguous reads, and low-quality reads (Q20 < 20), a total of 66,813,052 clean reads (total length of 6,681,305,200; 6.7 Gb) nucleotides were obtained. The Q20 (sequencing error rate < 1%) and GC percentages were 95.21 and 53.04%, respectively (Table 1).

**Table 1:** **Summary of Illumina Paired-end sequencing and assembly for *P. ternata***.

**Database**	**Number**	
Total clean reads	66,813,052	
Total length of clean reads (bp)	6,681,305,200	
Q20 percentage	95.21%	
GC percentage	53.04%	
**Assembly**	**Trinity**	**Bridger**
Number of contigs	120,983	
Total length of contigs (bp)	90,698,333	
Average length of contigs (bp)	750	
Max length of contigs (bp)	9170	
Min length of contigs (bp)	201	
Contig size N50 (bp)	1112	
Number of unigenes	89,068	93,451
Total length of unigenes (bp)	62,683,550	105,777,147
Average length of unigenes (bp)	703	1131
Max length of unigenes (bp)	9170	16,771
Min length of unigenes (bp)	201	100
Unigene size N50 (bp)	1078	1835

**Figure 2:**
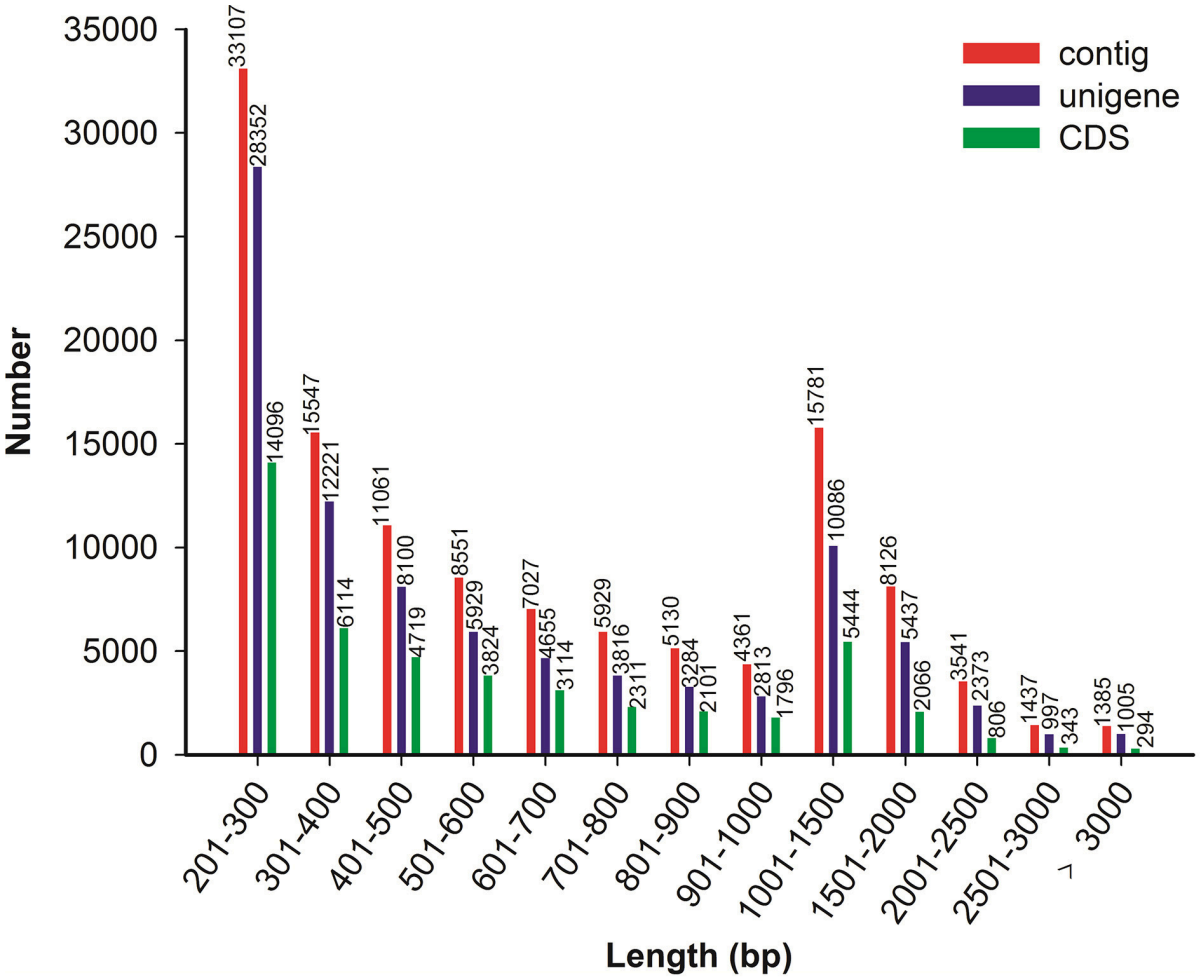
**Overview of the *P. ternata* transcripts assembly and the length distribution of the CDS**.

Because no reference genome exists for *P. ternata*, the reads were assembled *de novo*. Using the Trinity assembling program, clean reads were assembled into 120,983 contigs ranging from 201 to 9170 bp and with a mean length of 750 bp and an N50 length of 1112 bp. Among these contigs, 61,268 (50.64%) were longer than 500 bp, and 15,781 (13.04%) were longer than 1000 bp. Using paired-end joining and gap-filling methods, the contigs were further assembled into 89,068 unigenes with an average length of 703 bp and an N50 length of 1078 bp (Table 1). Among all unigenes, 19,898 (22.34%) were longer than 1000 bp, and 48,673 (54.64%) were less than 500 bp. In this study, we obtained a total of 51,642 CDSs and 9902 CDSs (19.17%) were longer than 1000 bp and 22,499 CDSs (43.57%) exceeded 500 bp. The length distributions of contigs, unigenes and CDSs are depicted in Figure 2 and the sequences of all unigenes assembled by Trinity are shown in Data Sheet 2.

Moreover, a new *de novo* transcriptome assembler, Bridger (version: r2014-12-01), was also used for assembly in our study (Chang et al., 2015). Compared with Trinity, more unigenes (93,451) with longer average length (1131 bp) and N50 length (1835 bp) were obtained by Bridger (Table 1). The sequences of all unigenes assembled by Bridger are shown in Data Sheet 3. Bridger can assemble more full-length reference transcripts, and reducing false positive transcripts in comparison with the state-of-the-art assemblers (Chang et al., 2015). Our results also indicated that Bridger is better than Trinity in transcriptome assembly in non-model plant, and the unigenes with longer average length will be helpful in cloning the full-length of cDNA and functional characterization of the genes. Nevertheless, Trinity is also a good assembler for transcriptome assembly from RNA-seq data without a reference genome (Grabherr et al., 2011; Bankar et al., 2015); the assembled results of Trinity are used for further analysis in this study.

To evaluate the quality and coverage of the assembled unigenes, all usable sequencing reads were realigned to the unigenes using SOAPaligner (Li et al., 2008), allowing up to 2 base mismatches. The sequencing depth ranged from 0.025 to 52,968-fold, with an average of 31.96-fold. About 62.17% of the unigenes were realigned by more than 10 reads, 22.87% were supported by more than 100 reads and 5.04% were supported by more than 1000 reads (Data Sheet 1: Figure S2). To assess the extent of transcript coverage provided by unigenes and to evaluate how coverage depth affected unigenes assembly, we plotted the ratio of assembled unigene length to *Oryza sativa* ortholog length against coverage depth (Data Sheet 1: Figure S3A). Although many of deeply covered *P. ternata* unigenes failed to cover the complete coding regions of their *O. sativa* orthologs, our unigenes covered most *O. sativa* ortholog coding regions. In our study, 2206 unigenes had ratios greater than 1, and 18,913 unigenes had ratios less than 1. Of note, to certain extent, increased coverage depth can result in higher coverage of the coding regions. The percentage of *O. sativa* ortholog coding sequences covered by all *P. ternata* unigenes was also measured and 3195 of the orthologs were covered by more than 80% of the unigenes and 2285 of the orthologs were covered by 40–80% of the unigenes. Around 45% of the orthologs were covered by only 20% or less (Data Sheet 1: Figure S3B) indicating that additional sequencing is essential for a more comprehensive coverage of the transcriptome of *P. ternata*.

### Functional annotation

To provide putative annotations of the assembled unigenes, all of the unigenes was subjected to a BLASTN search against the public protein databases (NR, Swiss-Prot, KEGG, COG) with *E* < 10^−5^. The unigenes were also searched against the *Arabidopsis* TAIR10 peptide database using the BLASTX algorithm with *E* < 10^−5^. Using this approach, ~53.33% of unigenes (47,504) were annotated in the five public databases (Data Sheet 1: Table S3). Among them, 4248 unigenes had significant matches in all five databases, 9753 unigenes annotated uniquely in NR database, 182 unigenes annotated uniquely in Swiss-Prot database, 8 unigenes annotated uniquely in COG database, 46 unigenes annotated uniquely in KEGG database, and there were no unigenes annotated uniquely in TAIR10 database (Data Sheet 1: Figure S4). The annotations of unigenes in all five databases are shown in Data Sheet 4.

Previous studies indicate that the longer sequences were more likely to obtain BLAST matches in the protein databases (Wang et al., 2010; Li et al., 2012; Liu et al., 2013), and this assumption was validated by our data which indicated that more than 83% of the unigenes larger than 1000 bp in length had BLAST matches in NR and Swiss-Prot protein databases. In contrast, only ~30% of unigenes shorter than 500 bp did (Data Sheet 1: Figure S5). The *E*-value distribution of the top hits in the NR database revealed that 46.11% of the mapped sequences had significant homology (*E* < 1e^−50^), and 17.80% of the sequences with greater than 80% similarity were found (Data Sheet 1: Figures S6A,C). The *E*-value and similarity distributions of the top hits in the Swiss-Prot database had comparable patterns with 36.41 and 15% of the sequences possessing significant homology and similarity, respectively (Data Sheet 1: Figures S6B,D). Our results also indicated that 35.79% of the unigenes had significant homology with sequences of *Vitis vinifera* (9011, 19.07%), followed by *Theobroma cacao* (6824, 14.44%), *O. sativa* (6158, 13.03%), and *Setaria italica* (3124, 6.61%) (Data Sheet 1: Figure S7). This suggests that the genome of *P. ternata* is more closely related to *V. vinifera* than to other model plant genomes.

### Gene ontology classification

**Figure 3:**
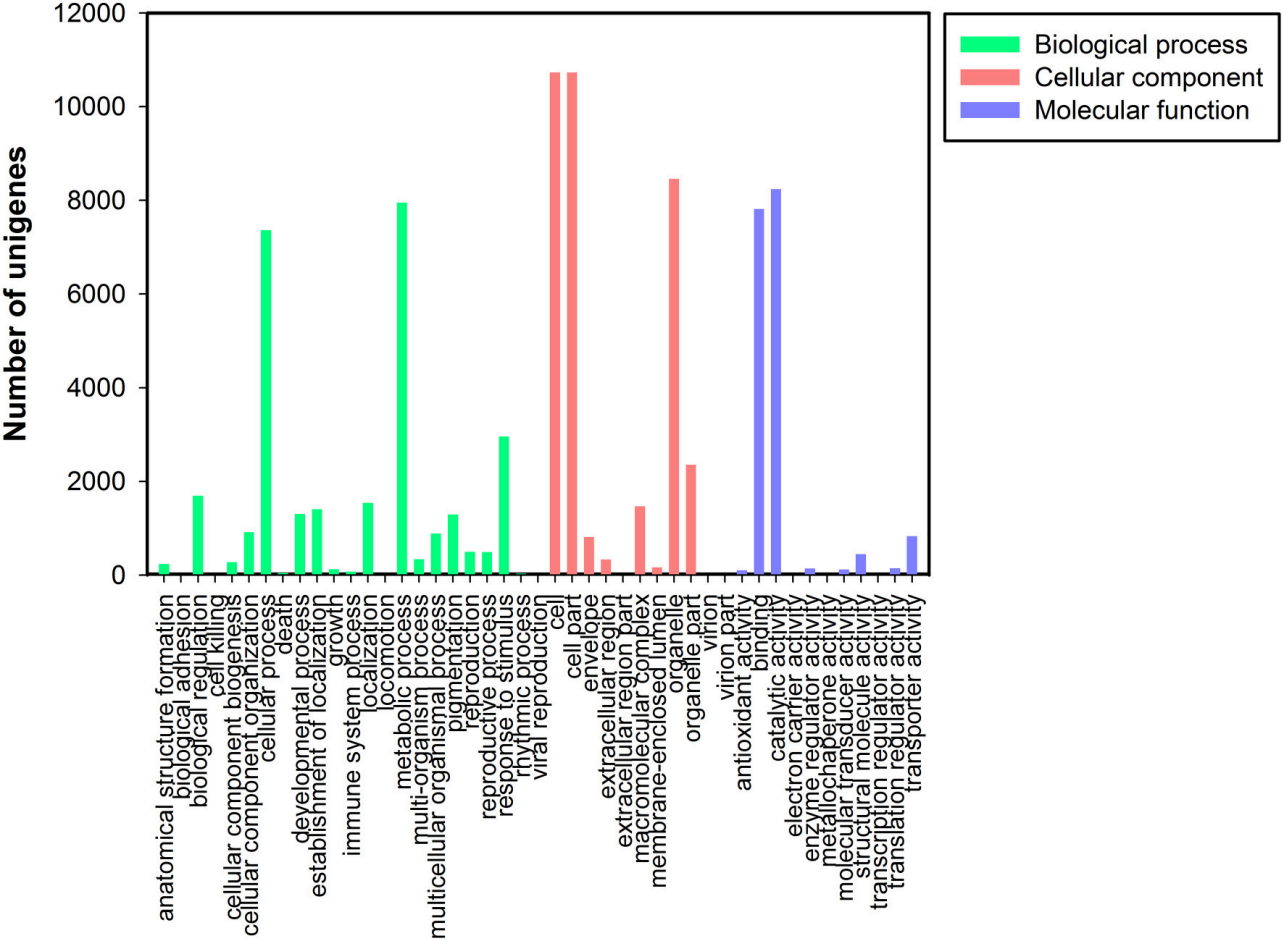
**Gene Ontology classification of assembled unigenes**. Total 16,671 unigenes were categorized into three main categories: biological process, cellular component, and molecular function.

Based on the NR annotation, Gene Ontology (GO) classification was used to classify the functions of all unigenes. Based on sequence homology, 16,671 unigenes were assigned to one or more ontologies, including 35,061 unigenes at the cellular component, 29,454 unigenes at the biological process, and 17,857 sequences at the molecular function (Figure 3). Within the cellular component category, cell (10,728, 30.60%), cell parts (10,728, 30.60%), and organelles (8457, 24.12%) represented the majorities. Under the biological process category, metabolic (7950, 26.99%) and cellular processes (7362, 24.99%) and response to stimulus processes (2959, 10.05%) were the most highly represented groups. Under the molecular function category, catalytic activity (8235, 46.12%) and binding (7816, 43.77%) were the most highly represented GO terms (Data Sheet 1: Table S4).

### Conserved domain annotation and COG classification

The conserved domains/families of the assembled unigenes encoding proteins were searched against the Pfam database (version 26.0) (Finn et al., 2014) using Pfam_Scan program. A total of 3601 protein domains were identified in 29,909 unigenes of *P. ternata* (Data Sheet 5). Among these protein domains/families, the pentatricopeptide repeat domain (PPR) was the most abundant domain type, found in 3204 unigenes. PPR-containing proteins are commonly found in plants and although their functions are still unclear, the PPR domain has been reported to exist in proteins involved in RNA editing (Fujii and Small, 2011; Shikanai and Okuda, 2011). Moreover, highly represented domains were the WD40 domain (1015 unigenes) and leucine-rich repeats (949 unigenes), which are primarily involved in protein-protein interactions (Kobe and Kajava, 2001). Then, a protein kinase domain (901 unigenes) was predicted in the derived transcriptomic sequences of *P. ternata*, which is involved in signal transduction pathways, development, cell division, and metabolism in higher organisms (Hanks and Quinn, 1991; Hanks and Hunter, 1995; Ahier et al., 2009). Other domains identified as being abundant included RNA recognition motifs (608 unigenes), protein tyrosine kinase (397 unigenes), reverse transcriptase (374 unigenes), mitochondrial carrier proteins (272 unigenes), and cytochrome P450s (271 unigenes). The 15 most abundant protein domains/families are represented in Data Sheet 1: Figure S8.

**Figure 4:**
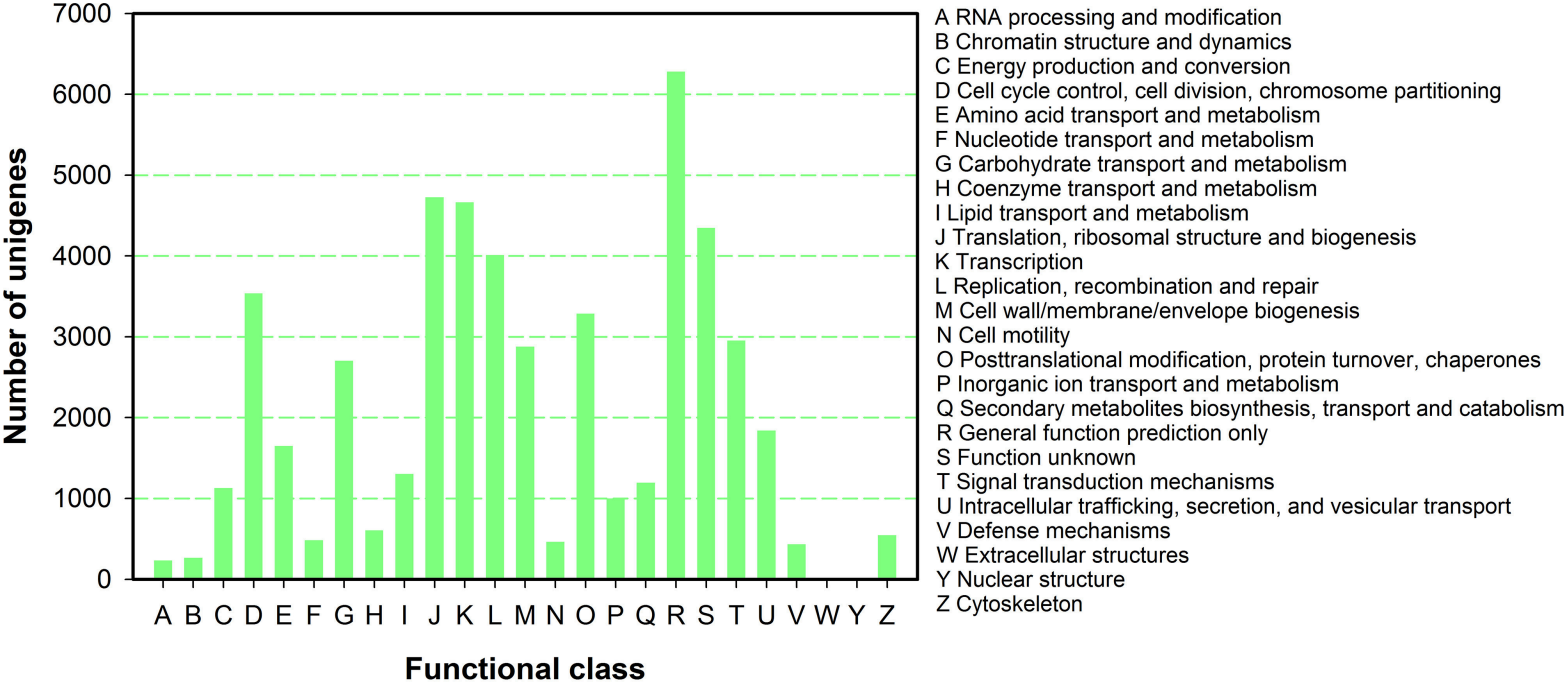
**COG function classification of *P. ternata* unigenes**.

To further predict gene function and to evaluate the completeness of the transcriptome library of *P. ternata*, all unigenes were subjected to a search against the COG database for functional prediction and classification. In total, 50,534 unigenes were annotated and grouped into 25 COG classifications (Figure 4). Among the 25 COG categories, the largest cluster was for general function prediction (6282, 12.43%), and this was followed by translation, ribosomal structure and biogenesis(4726, 9.35%), transcription (4661, 9.22%), unknown functions (4347, 8.60%), replication, recombination and repair (4009, 7.93%), and cell cycle control, cell division, and chromosome partitioning (3537, 7.00%), posttranslational modification, protein turnover, chaperones (3285, 6.50%), and signal transduction mechanisms (2955, 5.85%), and finally cell wall/membrane/envelope biogenesis (2878, 5.70%).

### Functional classification by KEGG

**Figure 5:**
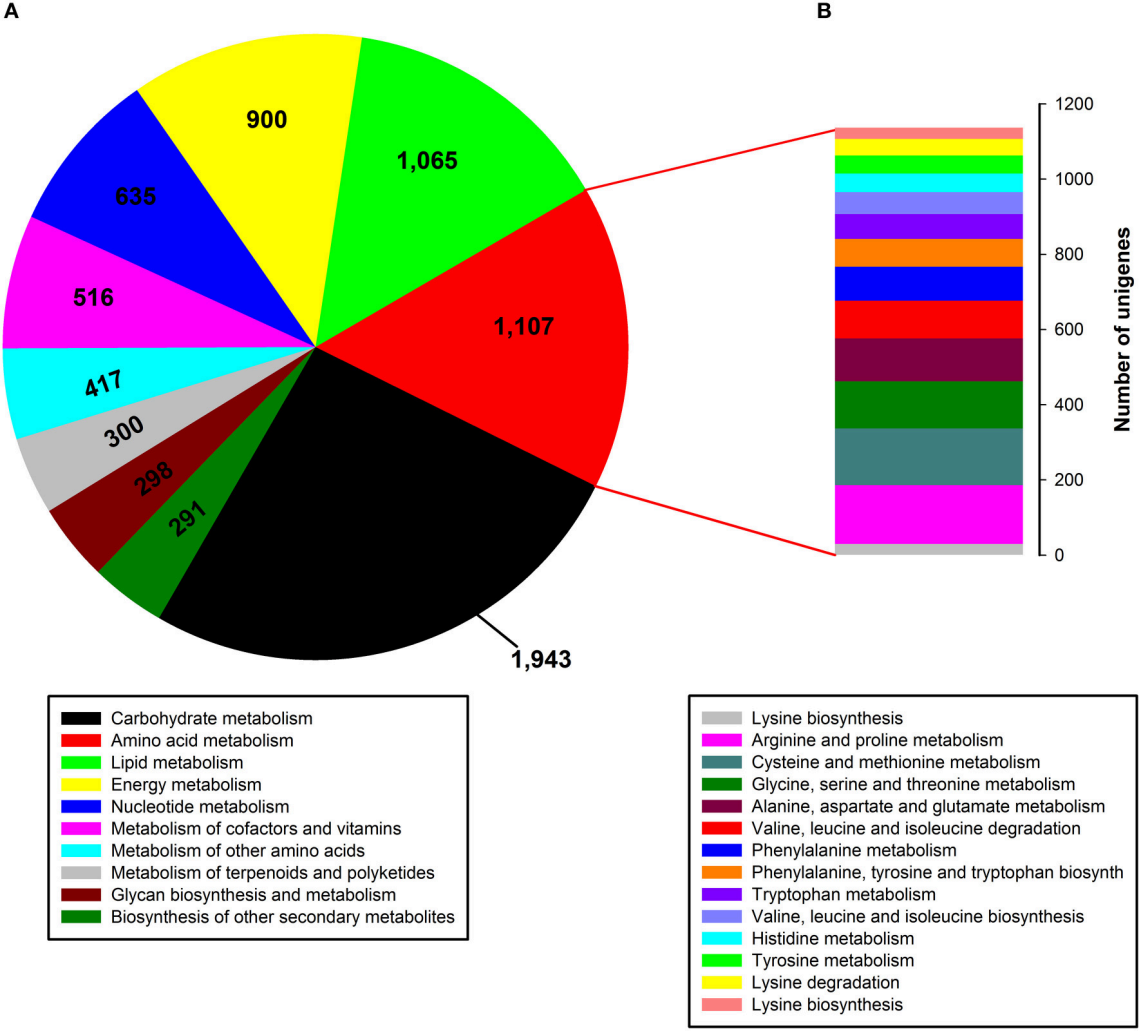
**Pathway assignment based on KEGG. (A)** Classification based on metabolism categories; **(B)** Classification based on amino acid metabolism categories.

To identify active biochemical pathways in *P. ternata*, unigenes were mapped to typical pathways in KEGG. Pathway-based analysis can help us understand the biological functions of genes. Based on a comparison against the KEGG database, a total of 13,899 unigenes (15.60%) were annotated in KEGG and 18,927 unigenes (21.25%) were assigned to 126 KEGG pathways (Data Sheet 1: Table S5). Among them, the metabolic pathway containing 3612 unigenes is the largest one, followed by biosynthesis of secondary metabolites (1673 unigenes), the ribosome (592 unigenes), RNA transport (590 unigenes), the spliceosome (441 unigenes), an mRNA surveillance pathway (394 unigenes), and plant-pathogen interaction (376 unigenes). The tubers grown in soil are more prone to pathogenic attack, so *P. ternata* needs an adequate supply of secondary metabolites to produce an adequate pathogen defense. Thus, 376 unigenes were assigned to the *plant-pathogen interaction* term as expected. In the metabolic pathway, the most represented subcategories were carbohydrate metabolism (1943 unigenes), followed by metabolism of amino acids (1107 unigenes), lipids (1065 unigenes), energy (900 unigenes), nucleotides (635 unigenes), cofactors and vitamins (516 unigenes), other amino acids (351 unigenes), terpenoids and polyketides (300 unigenes), and glycan biosynthesis and metabolism (298 unigenes), as well as biosynthesis of others secondary metabolites (291 unigenes) (Figure 5A). In the amino acid metabolism category, 14 subcategories comprised 1107 unigenes, the most represented categories were for metabolism of arginine and proline (156 unigenes), cysteine and methionine (151 unigenes), glycine, serine and threonine (125 unigenes), aspartate and glutamate (114 unigenes), and valine, leucine and isoleucine degradation (101 unigenes) (Figure 5B).

### Transcripts encoding enzymes involved in benzoic acid and ephedrine biosynthesis

Here, we focused on the discovery of genes involved in ephedrine and its precursor benzoic acid biosynthesis. Ephedrine biosynthesis in plants begins with L-phenylalanine, which is converted to *trans*-cinnamic acid by PAL. In *E. sinica*, at least four isoforms of *PAL* genes exist, and their expression in roots was higher than in aerial plant components (Okada et al., 2008). Similarly, in the transcriptome dataset of *P. ternata*, 6 unigenes annotated to *PAL* gene were identified (Table 2; Data Sheet 6).

**Table 2:** **Transcripts involved in ephedrine and benzoic acid biosynthesis in *P. ternata***.

**Gene name**	**EC number**	**Unigene numbers**
PAL, L-phenylalanine ammonia lyase	4.3.1.24	6
CNL, cinnamate: CoA ligase (AAE, acyl activating enzyme)	6.2.1.	1
CHD, cinnamoyl CoA hydratase-dehydrogenase (Belongs to the enoyl-CoA hydratase/isomerase family)	–	2
KAT, 3-ketoacyl-CoA thiolase (=3-oxo-3-phenylpropionyl-CoA thiolase)	2.3.1.16	4
BALDH, benzaldehyde dehydrogenase	1.2.1.7	1
AO4, aldehyde oxidase 4	1.2.3.1	4
CHY, 3-hydroxyisobutyryl-CoA hydrolase	3.1.2.4	12
PDC, pyruvate decarboxylase	4.1.1.1	8
AHAS, acetolactate synthase	2.2.1.6	10
BL, benzoate-CoA ligase	6.2.1.25	4

**Figure 6:**
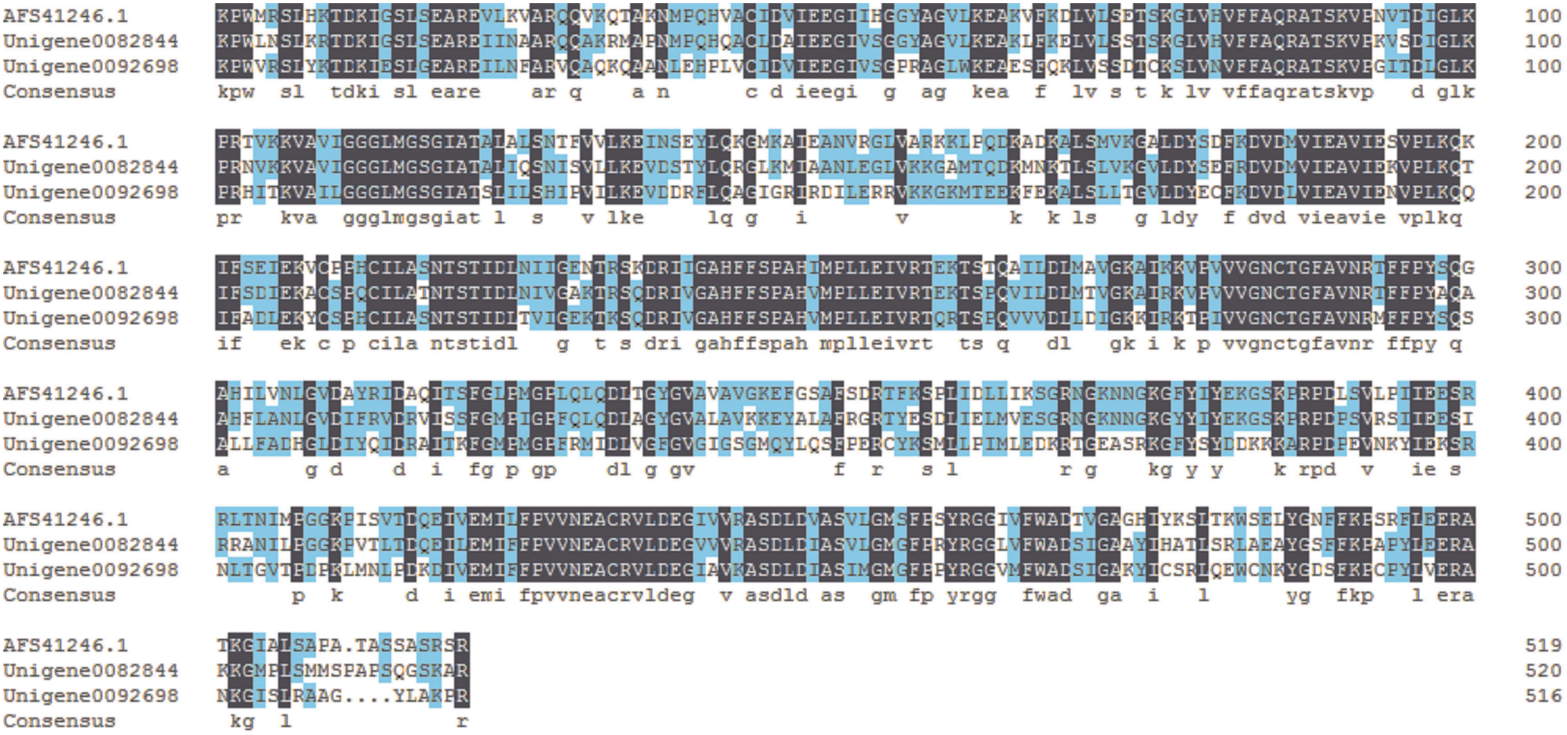
**Alignment of amino acid sequences of the putative *P. ternata CHD*s with Petunia hybrid *CHD* (AFS41246.1)**. Identical amino acid residues are shaded in dark blue. Light blue shade indicates 50% or more identity among all the aligned sequences.

Biosynthesis of benzoic acid from L-phenylalanine requires shortening of the side chain by two carbons, which can occur via the β-oxidative or non-β-oxidative pathways. The transcripts encoding enzymes in both of β-oxidative and non-β-oxidative pathways are found in the transcriptome dataset of *P. ternata*. One unique sequence was identified as with 74% similarity to *Hypericum calycinum CNL*, and four were annotated to the *KAT* gene (Table 2; Data Sheet 6). No unigene annotated to CHD gene was found, but 2 unigenes belong to the enoyl-CoA hydratase/isomerase family and showed high similarity (>75%) to petunia *CHD* gene (Qualley et al., 2012), suggesting that those might be the *CHD* candidate gene of *P. ternata* (Figure 6).

In this study, 1 unigene was identified as having 66% similarity to the *BALDH* gene of *A. thaliana*, and 5 unigenes were annotated to the *AO4* gene. Interestingly, 12 unigenes were annotated to *CHY* genes, which encoded 3-hydroxyisobutyryl-CoA hydrolase for catalyzing the conversion of cinnamoyl-CoA to benzaldehyde (Ibdah and Pichersky, 2009), suggested that benzaldehyde might come from cinnamoyl-CoA in *P. ternata*. Benzoate-CoA ligase (BL) catalyzes the formation of benzoyl-CoA from benzoate and CoA (Kliebenstein et al., 2007; Ibdah et al., 2009). In *P. ternata* transcriptome database, no unigene was annotated to *BL*, but we found 4 ungenes (unigene0092667, 0089644, 0080607, and 0044033) have close relationship to *Arabidopsis thaliana* BL (Genbank no. NP_176763.1) with the identities of 45–51%, respectively. The similar result was also found in *C. edulis* and *E. sinica* (Groves et al., 2015) (Table 2; Data Sheet 6). To the best of our knowledge, these putative *BALDH, AO4, CHY* and *BL* genes are first reported in *P. ternata*. Genes in the β-oxidative and non-oxidative pathways were identified, and we propose that benzoic acid biosynthesis in *P. ternata* likely occurs via both of these pathways.

ThPDC or AHAS might catalyze the condensation of pyruvic acid and benzoic acid to form 1-phenylpropane-1,2-dione (Müller et al., 2009), in the transcriptome of *P. ternata*, we identified 8 unigenes that may encode the *PDC* gene, and 10 unigenes were annotated as candidate *AHAS* genes Table 2; Data Sheet 6). After the formation of 1-phenylpropane-1,2-dione intermediate, there are three enzymes involved in ephedrine biosynthesis, viz. transaminases, reductases and *N*-methyltransferases (Figure 1). Up to now, no such enzymes and relevant genes in phenylpropylamino alkaloids biosynthesis were functionally characterized; only some candidates were predicted based on phylogenetic analysis (Groves et al., 2015). In *C. edulis* and *E. sinica* transcriptomes, the transaminases candidates closely related to aromatic amino acid transaminases or prokaryotic-type amino transferases (Groves et al., 2015). Unlikely, in *P. ternata* transcriptome database, 14 unigenes were annotated to transaminase, among them, 8 unigenes were annotated to alanine-glyoxylate transaminase, 4 unigenes were annotated to alanine transaminase, and 2 were annotated to ornithine-oxo-acid transaminase (Data Sheet 7). This result suggested the complexity of transaminase in phenylpropylamino alkaloids-producing plants. Moreover, 265 unigenes annotated to reductase (Data Sheet 8) and 211 unigenes annotated to *N*-methyltransferase (Data Sheet 9) were also found in *P. ternata* transcriptome database. Those results indicated that identifying the transaminase, reductase and *N*-methyltransferase in phenylpropylamino alkaloids biosynthesis should be very difficult and further research is needed.

### Relative expression levels of putative genes involved in ephedrine biosynthesis

**Figure 7:**
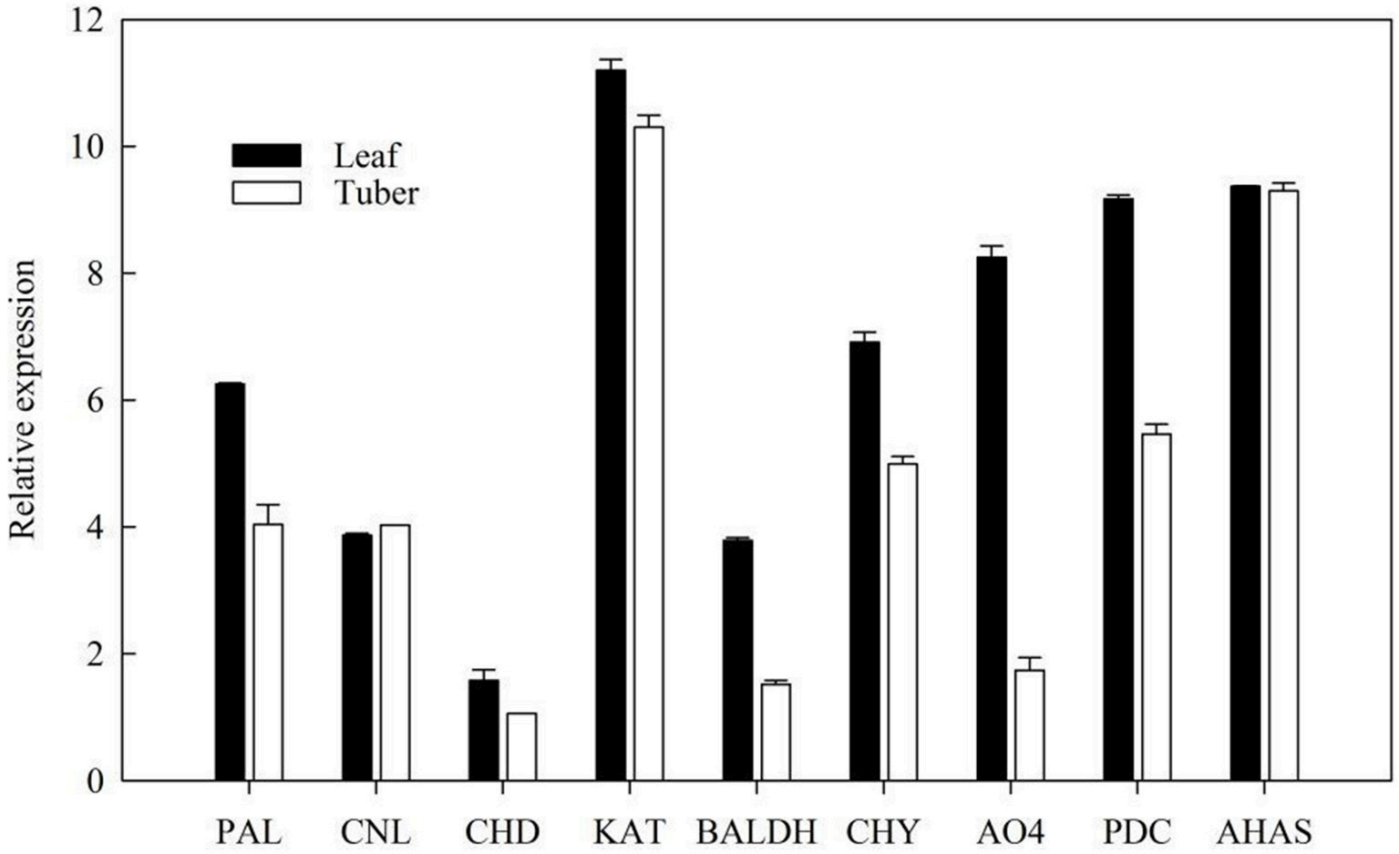
**Validation of candidate *P. ternata* unigenes involved in ephedrine biosynthesis by RT-qPCR**. Bars represent the mean (± SD) of three experiments.

The expression level of ephedrine related genes in the leaves and tubers of *P*. *ternata* were analyzed by RT-qPCR. The results showed that these genes exhibited different expression level in the leaves and tubers (Figure 7). All these genes were more highly expressed in the leaves than in tubers. Due to lack of detailed information concerning the downstream steps of ephedrine biosynthesis, the genes selected for RT-qPCR analysis were all located at the upstream of ephedrine biosynthesis. Therefore, our results were not unexpected, and it indicated that leaves may be the main organ for synthesizing the precursors of ephedrine. It is noteworthy that the transcript abundance of the *AHAS* gene was higher in the tubers as compared to that of PDC gene. Obviously, *AHAS* appears to have greater activity in the tubers. Although no more evidence is presently available, we suggested that *AHAS* was the major enzyme responsible for the biosynthesis of 1-phenylpropane-1,2-dione in *P. ternata*. We believe that these data will be helpful to further understand the mechanism of ephedrine biosynthesis.

### Identification of transcription factors involved in alkaloid biosynthesis

Transcription factors (TFs) play a crucial role in regulating the secondary metabolism, because they can regulate the expression of related genes at the transcriptional level to control the flux of secondary metabolites. The identification of such transcription factors will help us gain a better understanding of gene regulatory networks. Based on the BLASTX search against the Plant Transcription Factor Database database (PlnTFDB) (Pérez-Rodríguez et al., 2010), 1218 unigenes were annotated in 848 independent coding sequences of plant transcription factors (identity >80%) that belong to 68 known transcription factor families (Table 3 and Data Sheet 10). Among them, 79, 68, 67, 64, 57, and 53 unigenes were annotated to the *AP2-EREBP, bHLH, HB, MYB, C3H*, and *WRKY* families, respectively.

**Table 3:** **Statistics for putative transcription factors in *P. ternata* unigenes**.

**TF family**	**Number of unigenes**	**TF family**	**Number of unigenes**
*AP2-EREBP*	79	*NAC*	33
*bHLH*	68	*TRAF*	30
*HB*	67	*bZIP*	24
*MYB*	64	*SET*	24
*C3H*	57	*ARF*	22
*WRKY*	53	*Alfin-like*	22
*MYB-related*	53	*C2C2-GATA*	21
*CCAAT*	53	*GNAT*	20
*C2H2*	48	*C2C2-Dof*	19
*G2-like*	45	*MADS*	19
*Orphans*	42	Others	320
*SNF2*	35	Total number of TFs	1218

Some TFs are essential for alkaloid biosynthesis in plants. *AP2-EREBP* is a large super family of TFs, and it can be distinguished by containing one or two *AP2/ERF* DNA binding domain (Riechmann and Meyerowitz, 1998). In *Catharanthus roseus*, the *AP2/ERF*-domain transcription factor *ORCA2* and *ORCA3* in turn regulate a subset of alkaloid biosynthesis genes (van der Fits and Memelink, 2000; Zhang et al., 2011; Guo et al., 2013). In our study, 79 unigenes encoding *AP2-EREBP* transcription factors were found. Meanwhile, the *CrMYC2* transcription factor, a member of the *bHLH* family from *C. roseus*, may play a functional role in the regulation of expression of *ORCA* genes (Zhang et al., 2011). Besides, isoquinoline alkaloid biosynthesis in *Coptis japonica* is regulated by a unique *bHLH*-type transcription factor, *CjbHLH1* (Yamada et al., 2011). In present study, a total of 68 unigenes encoding *bHLH* TFs were identified. The strictosidine synthase (*STR*) contributes to the forming of strictosidine, the central intermediate leading to all monoterpenoid indole alkaloids. A *CrBPF1* transcription factor is similar to the MYB-like factor BPF1, and it appears to enhance elicitor-mediated *STR1* gene expression (Facchini and De Luca, 2008). In our database, 64 unigenes were annotated as *MYB* transcription factors. In *C. roseus, CrGBF1*, and *CrGBF2* are members of the *bZIP* TFs and participate in the regulation of expression of *STR* gene (Sibéril et al., 2001). A total of 24 unigenes coded for *bZIP* TFs have been identified. Transcription factors identified here may facilitate the research on the regulation of alkaloid biosynthesis in *P. ternata*.

### SSR detection and validation

SSRs are useful molecular genetic markers because of their relative abundance, and they have been widely applied for molecular-assisted selection (MAS) in plant breeding programs. Potential SSRs were detected in all of the 89,068 assembled unigenes using MISA software. A total of 14,468 SSRs were identified in 12,000 unigenes (Data Sheet 1: Table S6). Of all SSR-containing unigenes, 2053 sequences contained more than one SSR and 824 SSRs were present in compound form. SSRs derived from all unigenes are shown in Data Sheet 11. On average, we found 1 SSR per 19 Kb, which is similar to the frequency in cotton (1 SSR per 20 KB) (Cardle et al., 2000). Among the SSRs, the di-nucleotide repeat motifs were the most abundant types. SSRs with five tandem repeats were the most common (Data Sheet 1: Table S7). The most common type of di-nucleotide was AG/CT which accounted for 41.54% of the repeats, followed by AT/AT (5.04%) and AC/GT (4.29%). Among the tri-nucleotide repeats, both of CCG/CGG was the most frequent motifs (11.84%) (Data Sheet 1: Figure S9). Using Primer3, a total of 13,644 pairs of primers (Data Sheet 12) was designed and these SSRs could serve the foundation for future molecular breeding and genetic applications in this herb. To evaluate the amplification effect of these primer pairs, 20 primer pairs were randomly selected for the test. A total of 13 (65%) primer pairs successfully amplified the clear and repeatable bands, and 7 (35%) pair primers failed to generate PCR products. Among the 13 successful primer pairs, 10 primer pairs produced PCR amplicons at the expected size. However, 3 primer pairs generated PCR products longer than expected, and this may be due to the presence of introns. We believed that these unigene-derived SSR markers identified in our research will facilitate molecular genetics and molecular breeding in this valuable medicinal plant.

## Discussion

### Sequencing and annotation

In this study we performed RNA sequencing and report a *de novo* assembly of an important medicinal herb in China, *Pinellia ternata*, which particularly accumulates ephedrine in the tubers. In total, 89,068 unigenes were obtained with an average length of 703 bp and an N50 length of 1078 bp and we obtained a total of 51,642 CDSs and 9902 CDSs (19.17%) were longer than 1000 bp and 22,499 CDSs (43.57%) exceeded 500 bp.

NGS does not require a reference genome to gain the useful transcriptomic information, making this technology particularly useful for non-model organisms that often lack genomic sequence data (Strickler et al., 2012). In this study, the assembly results indicated that the length distribution pattern and mean contig and unigene lengths were similar to those in the previous studies of Illumina-transcriptome (Hao et al., 2011; Huang et al., 2012; Shu et al., 2013), suggesting that transcriptome sequencing data from *P. ternata* were effectively assembled. To our knowledge, this is the first report of large-scale transcriptome sequencing and analysis in *P. ternata* and these data offer abundant genomic information for *P. ternata*. In all of 89,068 unigenes, ~53.33% of unigenes (47,504) were annotated in the public databases. Furthermore, about 46.67% of unigenes (41,564) could not be matched to known genes, indicating that there is limited information about the genomes or transcriptomes of *P. ternata* and its related species.

In functional classification by KEGG, we found 91 and 71 unigenes were assigned to phenylalanine metabolism and phenylalanine, tyrosine and tryptophan biosynthesis, respectively (Figure 5B). The phenylalanine, tyrosine and tryptophan biosynthesis pathway is involved in L-phenylalanine synthesis, as a precursor common phenylpropylamino alkaloid biosynthesis. The phenylalanine metabolism pathway is involved in making 1-phenylpropane-1, 2-dione, as a putative precursor in the formation of phenylpropylamino alkaloids in plants (Krizevski et al., 2007). KEGG annotation data offer a valuable resource for investigating specific processes, functions, and pathways that will guide *P. ternata* research.

### Genes involved in ephedrine biosynthesis and expression analysis

It is believed that benzoic acid is the intermediate in the formation of phenylpropylamino alkaloids (Krizevski et al., 2010), so the genes in benzoic acid biosynthesis might also be involved in the biosynthesis of phenylpropylamino alkaloids. In *P. ternata* transcriptome database, the transcripts encoding enzymes involved in benzoic acid biosynthesis were found in this study, included both in β-oxidative or non-β-oxidative pathways, and two genes (*CHY* and *BL*) connecting the two pathways. But the main pathway and the functions of those genes in phenylpropylamino alkaloids biosynthesis are still far from elucidated. RT-qPCR has shown that the relative expressions of the genes involved in benzoic acid biosynthesis are highly expressed in the leaves, indicating that leaves may be the main organ for synthesizing the precursors of ephedrine. We also predicted that *AHAS* might be the major enzyme involved in 1-phenylpropane-1,2-dione biosynthesis in *P. ternata*.

## Conclusions

For the first time, this study provides the comprehensive data regarding the transcriptome of *P. ternata* and establishes *P. ternata* as an ideal herb for investigating ephedrine biosynthesis. We identified several candidate genes involved in ephedrine biosynthesis and these data can facilitate the study of molecular mechanisms of ephedrine synthesis as well as the engineering of microorganisms for *de novo* production of similar active ingredients. Our data can also be useful for molecular genetic research or genetic engineering as it represents the most abundant genetic resource for *P. ternata*, and will serve as a foundation for other functional genomics of *P. ternata* or closely related species.

Phenylpropylamino alkaloids are only produced in a few plant species. There is a lack of attention to their biosynthesis and only some candidate genes were predicted (Groves et al., 2015). This study also provided candidate genes in ephedrine biosynthesis in *P. ternata*. But the functions of those genes should be characterized in the future. Furthermore, the relationships between gene expression profiles and natural abundance of phenylpropylamino alkaloids also needs to be evaluated; this will help us understand the concentrations of pathway intermediates under steady-state flux and the accumulation phenylpropylamino alkaloid end-products.

## Author contributions

GZ and NJ designed the experiment, prepared samples for RNA-seq, and analyzed the data. WS and CM helped in data interpretation and manuscript preparation. SY and JC analyzed the data and performed RT-qPCR analysis, and GZ and JC prepared the manuscript. All authors read and approved the final manuscript.

### Conflict of interest statement

The authors declare that the research was conducted in the absence of any commercial or financial relationships that could be construed as a potential conflict of interest.

## Supplementary material

Data Sheet 1 **Supplementary figures and tables in this study**.

Data Sheet 2 **The unigene sequence assembled by Trinity (Fasta)**.

Data Sheet 3 **The unigene sequence assembled by Bridger (Fasta)**.

Data Sheet 4 **Annotation of *P. ternata* unigene**.

Data Sheet 5 **List of Pfam domain families**.

Data Sheet 6 **The main identified phenylpropylamino alkaloid biosynthetic genes from *P. ternata* unigenes**.

Data Sheet 7 **Transaminase discovery**.

Data Sheet 8 **Reductase discovery**.

Data Sheet 9 ***N*-methyltransferase discovery**.

Data Sheet 10 **Putative transcription factors encoding unigenes in *P. ternata***.

Data Sheet 11 **The information of SSR derived from all unigene**.

Data Sheet 12 **The sequences information of SSR primers**.
