# Supplementary material for: De novo Sequencing and Transcriptome Analysis of Pinellia ternata Identify the Candidate Genes Involved in the Biosynthesis of Benzoic Acid and Ephedrine
Source: Front Plant Sci. 2016 Aug 16;7:1209. doi: 10.3389/fpls.2016.01209 (PMC4986801; doi:10.3389/fpls.2016.01209)
Supplement: Data Sheet 1 — Supplementary figures and tables in this study. [file DataSheet1.PDF]

Data Sheet 1. Supplementary Figures and Tables for this study.

Figure S1. The picture of *P. ternate*.

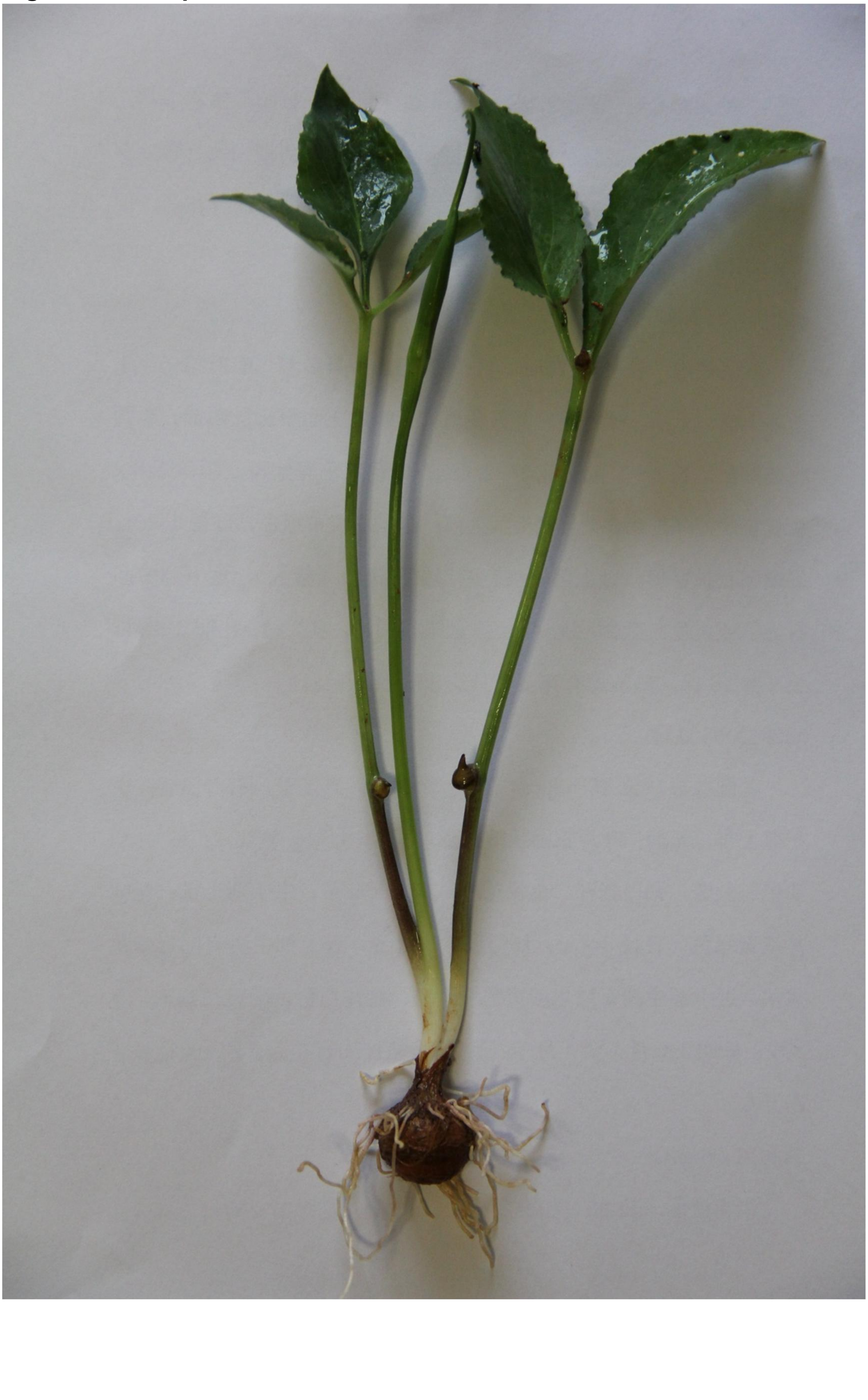

Figure S2. Assessment of assembly quality. Distribution of unique-mapped reads of the assembled unigenes.

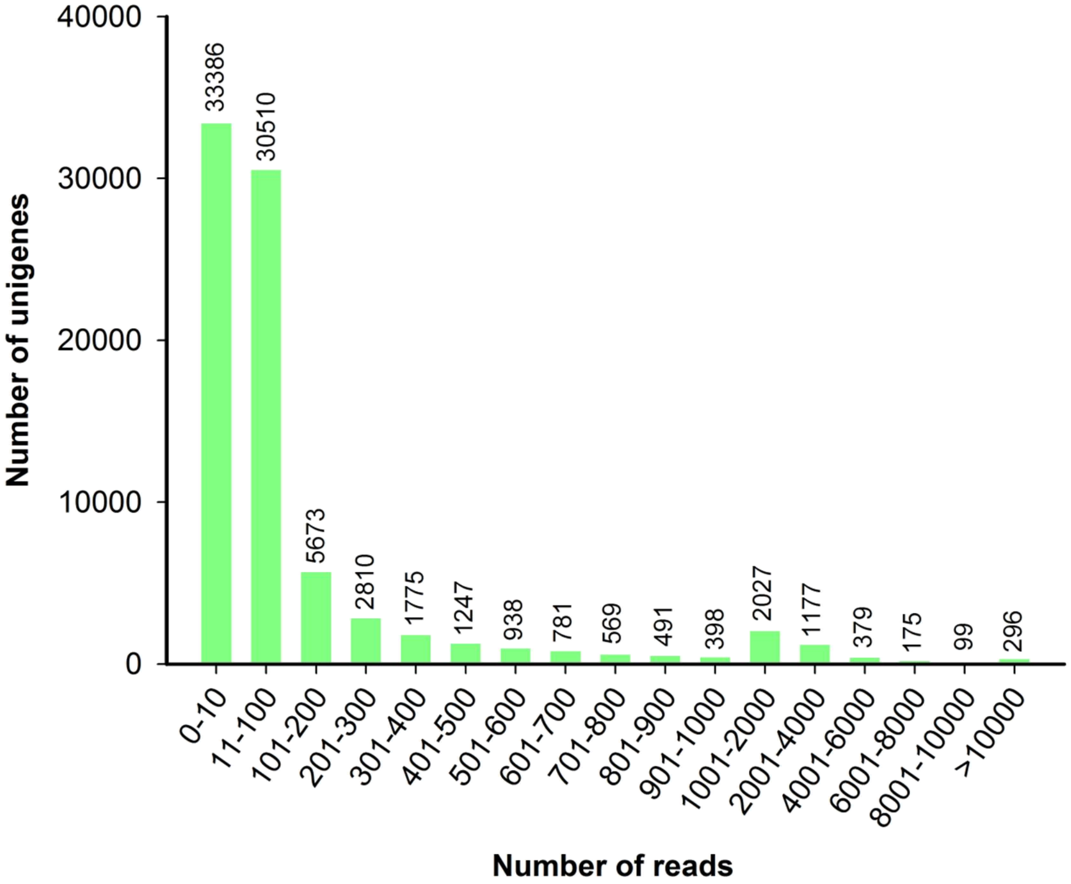

Figure S3. Comparison of *P. ternata* unigenes to orthologous *O. sativa* coding sequences. (A) The ratio of *P. ternata* unigene length to *O. sativa* ortholog length was plotted against *P. ternate* unigene coverage depth. (B) Total percent of *O. sativa* ortholog coding sequence that was covered by all *P. ternata* unigenes.

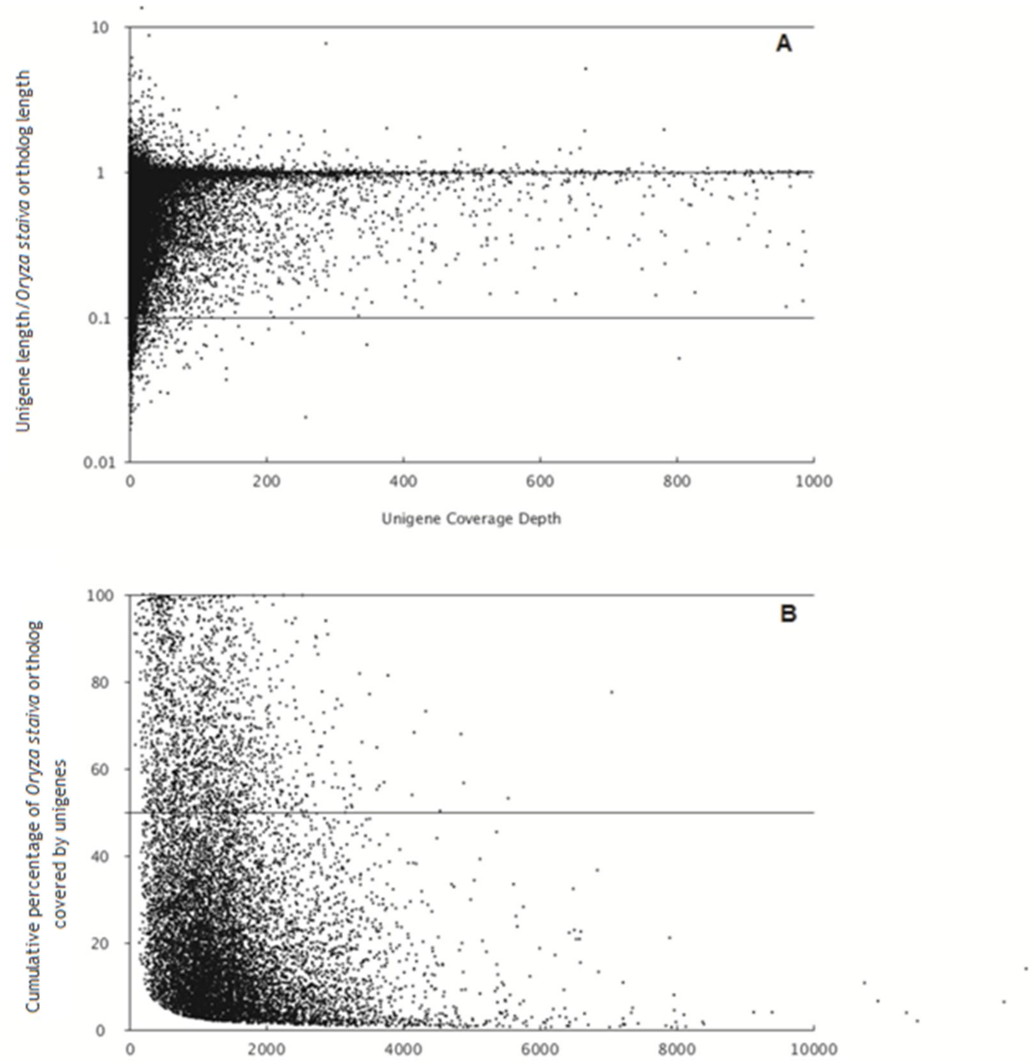

Figure S4. Venn diagram results from diverse databases. Venn diagram of number of unigenes annotated by BLASTX with an E-value threshold of 10^{-5} against protein databases. The numbers in the circles indicate the number of unigenes annotated by single or multiple databases.

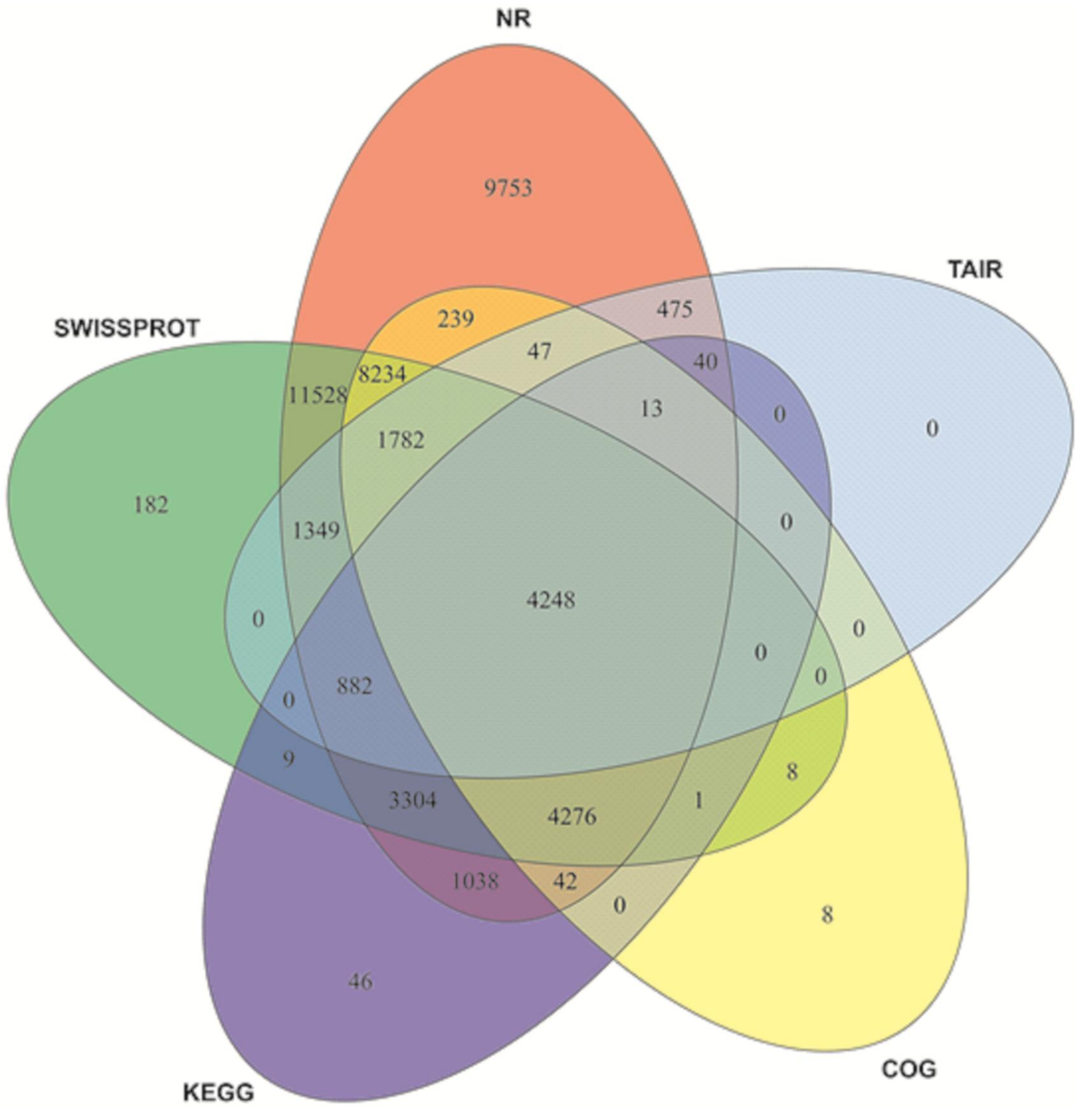

Figure S5. Comparison of unigene length between hit and nohit unigenes. (A) Comparison of unigene length between hit and no hit unigenes in the Nr databases. (B) Comparison of unigene length between hit and no hit unigenes in the Swiss-prot database. Longer contigs were more likely to have BLASTx homologs in protein database. In this study, more than 83% of unigenes over 1000 bp in length had BLAST matches, whereas only less than 30% of unigenes shorter than 500bp did.

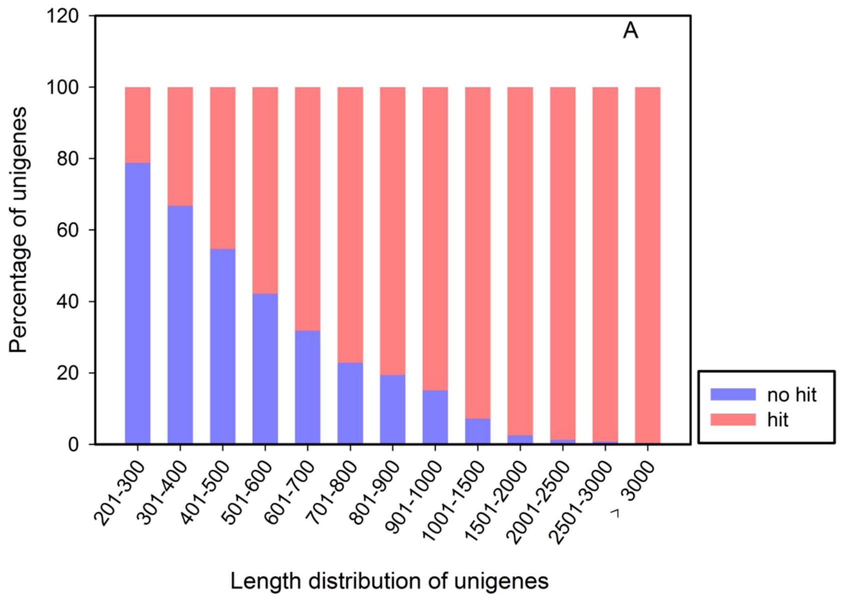

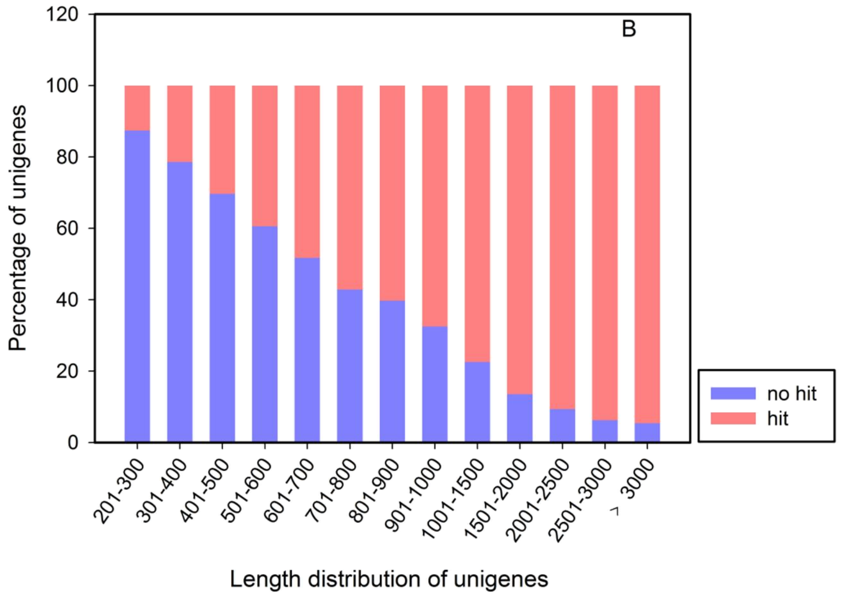

Figure S6. Characterization of searching the assembled unigenes against NCBI NR and Swiss-Prot protein databases. (A) E-value proportional frequency distribution of BLAST hits against the Nr database. (B) E-value proportional frequency distribution of BLAST hits against the Swiss-Prot database. (C) Similarity distribution of the top BLAST hits for the assembled unigenes with a cutoff of 1E-5 in Nr database. (D) Similarity distribution of the top BLAST hits for the assembled unigenes with a cutoff of 1E-5 in Swiss-Prot database.

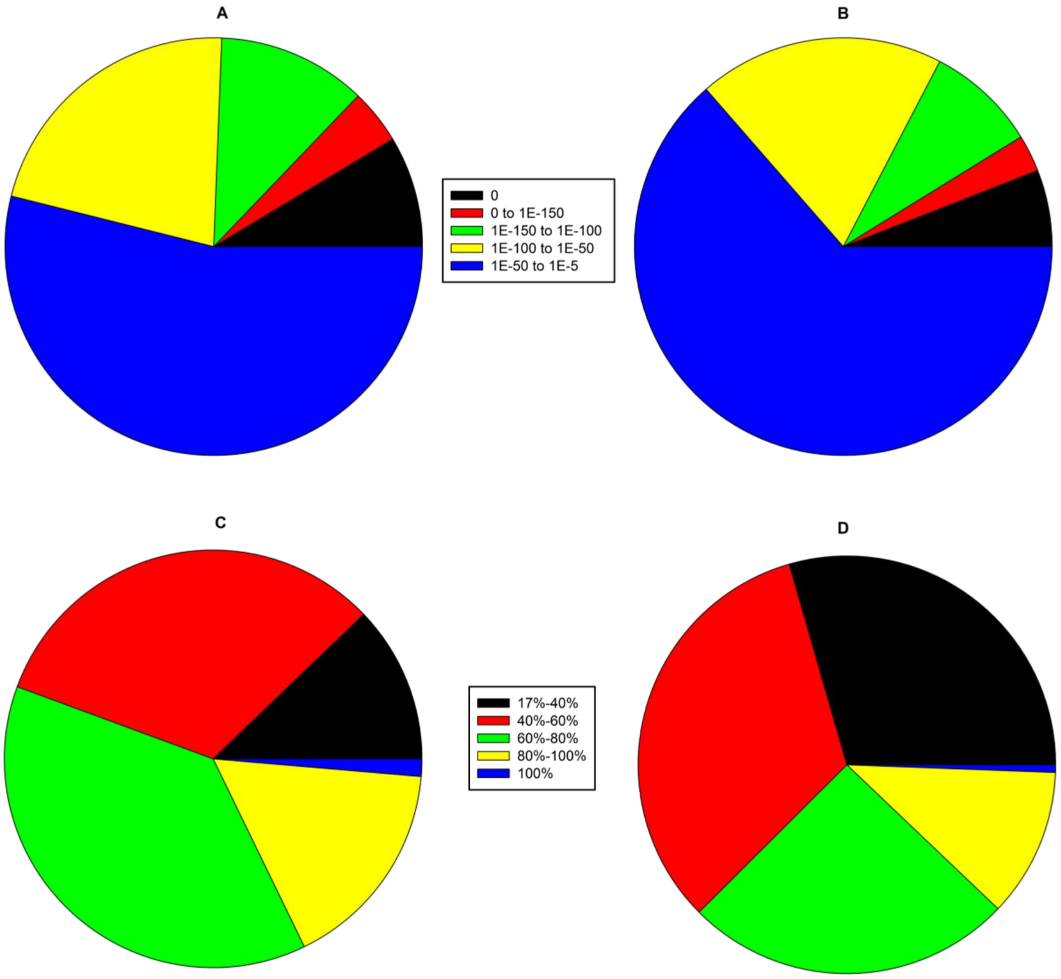

Figure S7. Top-hit species distribution for sequences from *P. ternate* submitted BLASTX against the NCBI-Nr database.

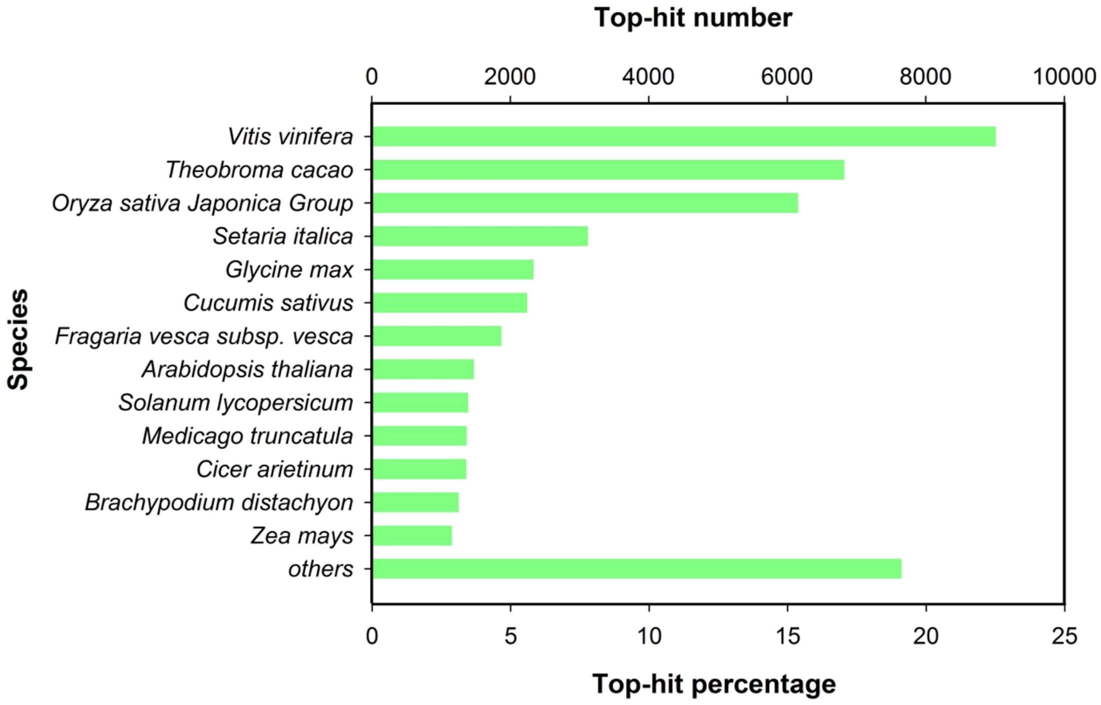

Figure S8. Top 15 Pfam domains/families predcted in *P. ternate* unigenes.

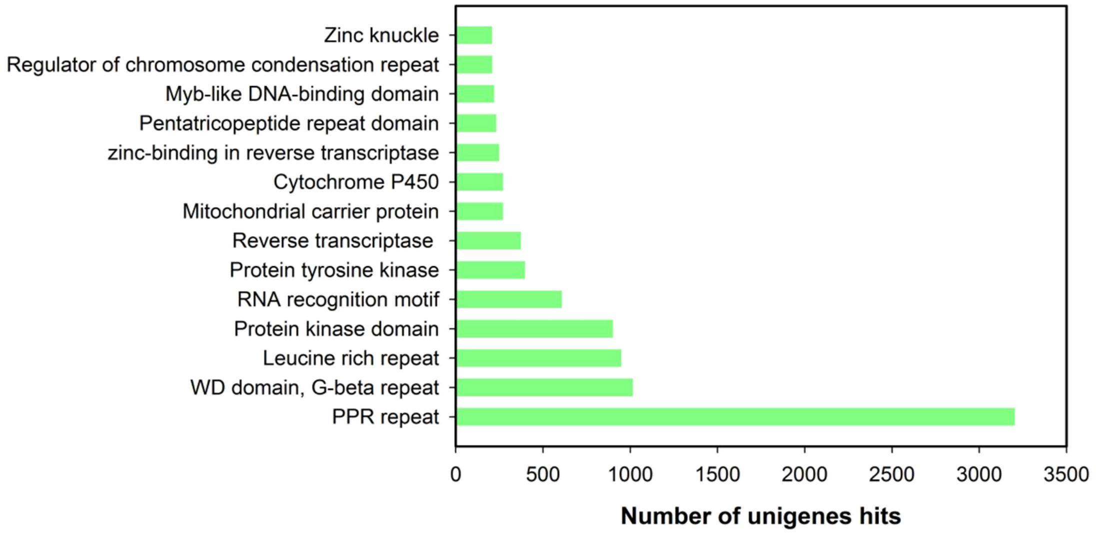

Figure S9. Frequency distribution of SSRs based on motif types. The AG/CT di-nucleotide repeat motif was the most abundant motif detected.

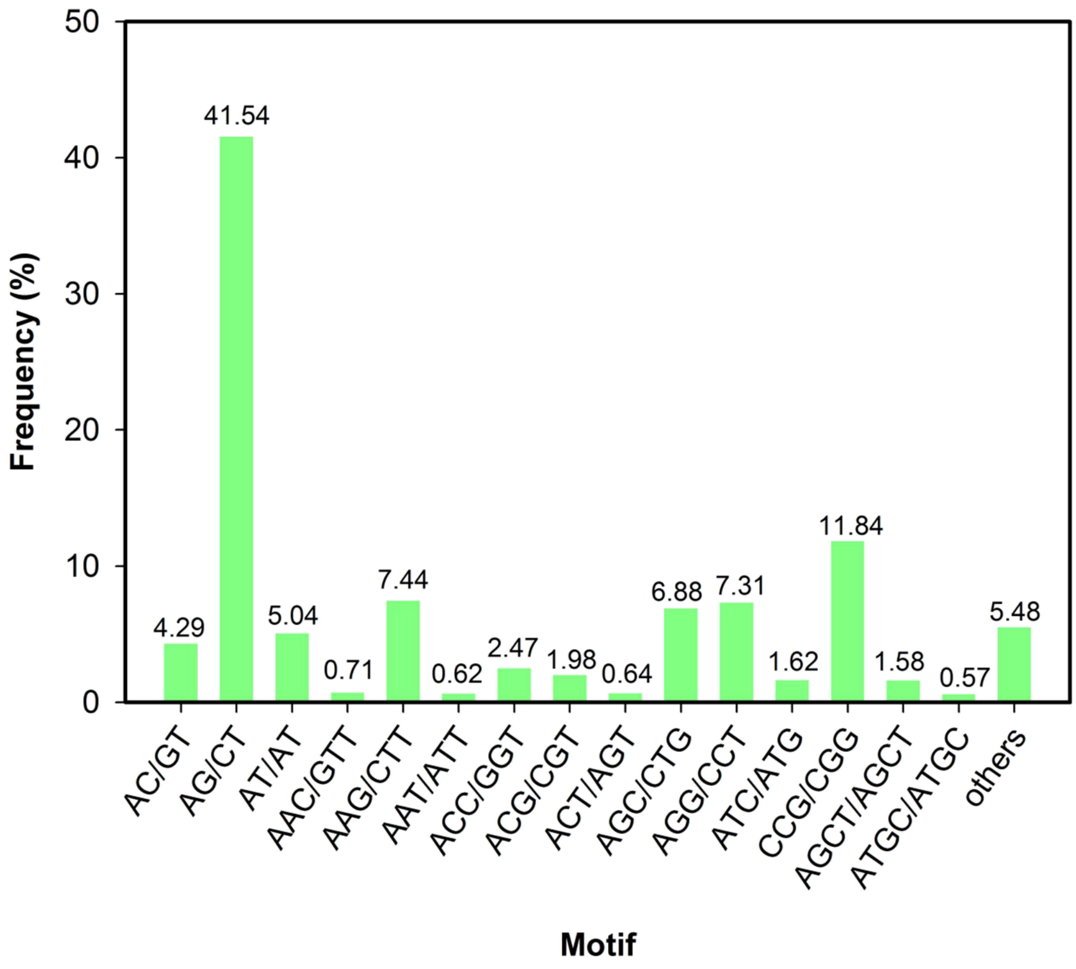

Table S1. Primers used for gene expression analysis by RT-qPCR.

Genes	Unigenes	Annotation	Primers sequences (5'-3')
PAL	Unigene0085432	phenylalanine ammonia-lyase	F: GTCCTGCTTCGGCTTCGTCA<br>R: CCAACATCCTGGCTCTGCTCTC
CNL	Unigene0047473	cinnamate: CoA ligase	F: GTGGTGCCAACTTTGTGCCC<br>R: GATGGAGGTGCGTTGGGAGT
CHD	Unigene0092698	enoyl-CoA hydratase/isomerase family	F: GTCTCGGTGTAGGCGCTCT<br>R: GAGTTCACGGGAGGGTTCA
KAT	Unigene0035555	3-ketoacyl-CoA thiolase (=3-oxo-3-phenylpropionyl-CoA thiolase)	F: TGATCCTGTTCTTGGCGTGTA<br>R: CTTGGGAGGGGTCAATAAATC
BALDH	Unigene0058009	benzaldehyde dehydrogenase	F: ACCTGATGGGACTTTTGCTT<br>R: TTCCATGAGGACAACTGCTT
CHY	Unigene0074170	3-hydroxyisobutyryl-CoA hydrolase	F: CCATCCTATCCTTTCGCTGAC<br>R: TGCTGGGGCTTCCTTCTATCT
AO4	Unigene0077151	aldehyde oxidase 4	F: GGTGCAGCCATAAGTGAAGT<br>R: CCATCTGAGTTTGCGAGGTA
PDC	Unigene0052431	pyruvate decarboxylase	F: AGGGCGGTGTAGTTCCAGTTC<br>R: CGGCGAGTTGCGGATATGAGT
AHAS	Unigene0053981	acetolactate synthase	F: TGTCCTCTTCTCCGACTCCA<br>R: GCTCGTTGGGACCGTATCT

Table S2. SSR primer pairs validated in this study.

Name	(5'-3')	Size	Unigene
PtSSR01F	GTGCTTCAGGAGGACCGTAG	150	Unigene0000197
PtSSR01R	CACCTCCCGGAAAAAGAGA		
PtSSR02F	GAAGATCCTCGTGTGGAAGG		Unigene0000456
PtSSR02R	CACGCAGCTGTTCGTTAAAA	128	
PtSSR03F	AAAGCTCGATCGGACAAGAA		Unigene0000800
PtSSR03R	TCTTTACCTCACCACCCTGC	152	
PtSSR04F	CCAATAGCCAGAGAGTGTGTAGG		Unigene0000709
PtSSR04R	TGTTAATTGTGTCGCCCAAA	192	
PtSSR05F	GGATTCTACGGTCCGTCTCC	218	Unigene0000202
PtSSR05R	TGGATCACGCTCCACAATAA		
PtSSR06F	GTCGGGAGAGGAGATCACAG	183	Unigene0000429
PtSSR06R	CCTGCTCGCTTTCTCTGAGT		
PtSSR07F	GCGCAGAAGAAGGAAAACAA	106	Unigene0000478
PtSSR07R	TAACCTCTCCCTCACTCCCC		
PtSSR08F	CACCCCGTTCGACAATAATC	270	
PtSSR08R	GACAATTCGCCCCAACTCTA		Unigene0000517
PtSSR09F	GTTTCCTCCGTTGTTGCCTA	258	Unigene0000782
PtSSR09R	ACGTGCCTCTGATTTCTGCT		
PtSSR10F	ATGAGTCAAAATGGCGCTCT	243	Unigene0001209
PtSSR10R	CAAAGACGACAGCTCCACAA		
PtSSR11F	TCGAACCAGACCATCCTTTC	134	Unigene0001675
PtSSR11R	GTGCCTCGATCGATCACTTT		
PtSSR12F	TGTGCACAATAGCACTTCGTC	109	Unigene0001739
PtSSR12R	TTTGTCCCTCAAAATCCTGG		
PtSSR13F	CATTGATGGCTTCCCCTCTA	182	Unigene0000022
PtSSR13R	GGGTGGATGACAACCAACTT		
PtSSR14F	AGTGTTTGAGGCTTGGGATG	242	Unigene0004757
PtSSR14R	AGATCCCGGTTGCTTGTATG		
PtSSR15F	ATCAATATGCAAGCCCAAGC	101	Unigene0007242
PtSSR15R	GGGCTTTAGTCGGGCTTTAC		
PtSSR16F	TCTAATGCAATAAAATCGACTCAG	124	Unigene0008371
PtSSR16R	TCCATTCCATTCGACTCCAT		
PtSSR17F	ATCTCACCCTCGGCTGACTA	256	Unigene0012561
PtSSR17R	TGAGGAAGATGGGATGGAAG		
PtSSR18F	GGTCTCCGCAGAGTAGATCG	210	Unigene0019855
PtSSR18R	GGTGTTTGCAAAGCTCTCG		
PtSSR19F	CCACGTCTGAGCCGATAGAT	269	Unigene0042362
PtSSR19R	CCCAGACGAAGCAGAAAAAG		
PtSSR20F	CAACACAACAGCACATGCAC	255	Unigene0022705
PtSSR20R	TCTCCATCTCGGGTTTCATC		

Table S3. Summary of annotation percentages of *P. ternata* unigenes compared to public databases

Database	Number of unigenes	Annotation percentage (%)
NR	47,250	53.05
SwissProt	35,803	40.20
KEGG	13,899	15.60
COG	18,898	21.22
TAIR10	8,836	9.92
All annotated	47,504	53.33
Total unigenes	89,068	

Table S4. Gene Ontology classification

Gene Ontology	Class	Number of Unigene
**Biological process**	anatomical structure formation	235
	biological adhesion	2
	biological regulation	1,691
	cell killing	2
	cellular component biogenesis	276
	cellular component organization	916
	cellular process	7,362
	death	46
	developmental process	1,301
	establishment of localization	1,403
	growth	127
	immune system process	73
	localization	1,544
	locomotion	16
	metabolic process	7,950
	multi-organism process	336
	multicellular organismal process	885
	pigmentation	1,291
	reproduction	501
	reproductive process	493
	response to stimulus	2,959
	rhythmic process	37
	viral reproduction	8
**Cellular component**	cell	10,728
	cell part	10,728
	envelope	815
	extracellular region	335
	extracellular region part	9
	macromolecular complex	1,468
	membrane-enclosed lumen	164
	organelle	8,457
	organelle part	2,355
	Virion	1
	virion part	1
**Molecular function**	antioxidant activity	99
	binding	7,816
	catalytic activity	8,235
	electron carrier activity	19
	enzyme regulator activity	140
	metallochaperone activity	2
	molecular transducer activity	119
	structural molecule activity	445

	transcription regulator activity	5
	translation regulator activity	148
	transporter activity	829
**Total**		**82,372**

Table S5. Mapping of *P. ternata* unigenes to KEGG biochemical pathways.

KEGG categories represented	No. of uniques
**Metabolism**	**7,472**
Carbohydrate metabolism	1,943
Energy metabolism	900
Lipid metabolism	1,065
Nucleotide metabolism	635
Amino acid metabolism	1,107
Metabolism of other amino acids	417
Glycan biosynthesis and metabolism	298
Metabolism of cofactors and vitamins	516
Metabolism of terpenoids and polyketides	300
Biosynthesis of other secondary metabolites	291
**Genetic Information Processing**	**4,382**
Folding, sorting, and degradation	1,218
Replication and repair	450
Translation	2,061
Transcription	653
**Environmental Information Processing**	**468**
Membrane transport	11
Signal transduction	457
**Cellular Processes**	**687**
Transport and catabolism	687
**Organismal Systems**	**516**
Environmental adaptation	469
Immune system	47
**Unassigned[a]**	**5,285**

[a] Unassigned unique sequences are those that have significant similarities to known sequences in the KEGG database, but whose functions in biochemical pathways are unclear.

Table S6. Summary of SSR searching results.

Item	Number
Total number of sequences examined	89,068
Total size of examined sequences (bp)	62,683,550
Total number of identified SSRs	14,468
Number of SSR containing sequences	12,000
Average number of SSRs per 19 Kb	1
Number of sequences containing more than 1 SSR	2,053
Number of SSRs present in compound formation	824

Table S7. Distribution of identified SSRs using the MISA software

Motif	Repeat numbers									total	%
	4	5	6	7	8	9	10	11	12		
Di-	0	0	1,583	1,230	1,338	1,896	1,125	226	3	7,401	51.15
Tri-	0	3,524	1,792	637	51	0	0	0	55	6,004	41.50
Tetra-	617	169	32	0	0	0	0	0	0	818	5.65
Penta-	110	8	0	0	0	0	0	0	0	118	0.82
Hexa-	127	0	0	0	0	0	0	0	0	127	0.88
Total	854	3,701	3,407	1,867	1,389	1,896	1,125	226	3	14,468	100
%	5.90	25.58	23.55	12.90	9.60	13.10	7.78	1.56	0.02	100	
